# Inflammatory macrophages interrupt osteocyte maturation and mineralization via regulating the Notch signaling pathway

**DOI:** 10.1186/s10020-022-00530-4

**Published:** 2022-09-04

**Authors:** Shengfang Wang, Lan Xiao, Indira Prasadam, Ross Crawford, Yinghong Zhou, Yin Xiao

**Affiliations:** 1 School of Mechanical, Medical and Process Engineering, Faculty of Engineering, Queensland University of Technology, Brisbane, QLD 4000 Australia; 2 Centre for Biomedical Technologies, Queensland University of Technology, Brisbane, QLD 4000 Australia; 3 Australia-China Centre for Tissue Engineering and Regenerative Medicine, Brisbane, QLD 4000 Australia; 4 School of Dentistry, Faculty of Health and Behavioural Sciences, The University of Queensland, Brisbane, QLD 4006 Australia

**Keywords:** Osteocytes, Matrix mineralization, Macrophages, Notch signaling pathway

## Abstract

**Background:**

It is well-known that both macrophages and osteocytes are critical regulators of osteogenesis and osteoclastogenesis, yet there is limited understanding of the macrophage-osteocyte interaction, and how their crosstalk could affect bone homeostasis and mineralization. This research therefore aims to investigate the effects of macrophage polarization on osteocyte maturation and mineralization process.

**Methods:**

A macrophage-derived conditioned medium based osteocyte culture was set up to investigate the impact of macrophages on osteocyte maturation and terminal mineralization. Surgically induced osteoarthritis (OA) rat model was used to further investigate the macrophage-osteocyte interaction in inflammatory bone remodeling, as well as the involvement of the Notch signaling pathway in the mineralization process.

**Results:**

Our results identified that osteocytes were confined in an immature stage after the M1 macrophage stimulation, showing a more rounded morphology, higher expression of early osteocyte marker E11, and significantly lower expression of mature osteocyte marker DMP1. Immature osteocytes were also found in inflammatory bone remodeling areas, showing altered morphology and mineralized structures similar to those observed under the stimulation of M1 macrophages in vitro, suggesting that M1 macrophages negatively affect osteocyte maturation, leading to abnormal mineralization. The Notch signaling pathway was found to be down regulated in M1 macrophage-stimulated osteocytes as well as osteocytes in inflammatory bone. Overexpression of the Notch signaling pathway in osteocytes showed a significant circumvention on the negative effects from M1 macrophage.

**Conclusion:**

Taken together, our findings provide valuable insights into the mechanisms involved in abnormal bone mineralization under inflammatory conditions.

**Supplementary Information:**

The online version contains supplementary material available at 10.1186/s10020-022-00530-4.

## Background

Bones are critical parts of vertebrate animals building the skeletal system, which supports our body structure, protects important organs, and provides leverage and movement for many important physiological activities (Naili et al. 2011). Bones are not static tissues and constantly remodel during our lifetime and it is the balance between bone formation and bone resorption that maintains a stable skeletal structure (Naili et al. 2011).

The bone formation process is achieved by osteoblasts (Nanes 2016) which are originally differentiated from bone marrow-derived mesenchymal stem cells (MSCs) (Franz-Odendaal et al. 2006). Bone resorption is achieved by osteoclasts which are derived from macrophages from the immune system (Choi et al. 2016). The differentiation process from MSCs to osteoblasts is regulated by both the skeletal and immune systems (Nanes 2016; Horwood 2016; Taylor et al. 2007). Immune cell-derived cytokines can significantly regulate activities of osteoblasts (Wei et al. 2018). Among immune cells, macrophage is well-studied and believed to play an important role in regulating bone remodeling. Macrophage is not only the precursor of the osteoclast, but also its regulator. During macrophage polarization, major phenotypes, M1 or M2 subtypes can produce various cytokines and regulate osteoclast activities (Horwood 2016).

Some osteoblasts continue to differentiate towards osteocytes and bury themselves in the bone matrix (Franz-Odendaal et al. 2006). On the other hand, osteocytes can also regulate the differentiation of osteoblasts with mature osteocytes producing sclerostin (SOST), a regulatory protein which can prohibit osteoblast differentiation (Nanes 2016; Tu et al. 2012). The differentiation of osteoclasts requires the receptor activator of nuclear factor-κ B Ligand (RANKL) (Kikuta et al. 2016). Osteocytes are the major producer of RANKL, and are also the major producer of its inhibitor, osteoprotegerin (OPG) (O'Brien et al. 2013). Dysfunction of osteocytes has been observed in certain bone related diseases, especially osteoarthritis (OA) (Jaiprakash et al. 2012). As the phenotype of osteocytes switched in OA subchondral bone, the nano structure of bone mineralization was altered (Zuo et al. 2016).

Both macrophages and osteocytes are regulators of bone formation and bone resorption, but there is a limited understanding of the interactions between macrophages and osteocytes. It will be interesting to know whether the interaction exists and whether the crosstalk within macrophages and osteocytes could affect bone mineralization. The status of osteocytes may greatly affect the process of bone mineralization, and subsequently the bone quality. Macrophages have a long-life cycle and play important roles in chronic inflammatory reactions (Sima and Glogauer 2013). Associated with the facts that, phenotype of osteocytes was switched in chronic inflammatory condition (Jaiprakash et al. 2012), it is unknown if there is a correlation between macrophages and osteocyte dysfunction in chronic inflammatory bone diseases. Using an indirect co-culture model based on the macrophage-derived conditioned medium, the present study investigated the effect of macrophages on osteocyte maturation. Chronic inflammatory subchondral bones from surgically induced OA rat model were further applied to observe the correlation between macrophage polarization and mineralization.

## Methods

### Macrophage conditioned medium collection

RAW264.7, a widely used murine macrophage cell line (Gao et al. 2015; Wang et al. 2017), was cultured in Dulbecco's Modified Eagle Medium (DMEM; Life Technologies, USA) with 1% penicillin and streptomycin (P/S), 10% fetal bovine serum (FBS). 10 ng/mL lipopolysaccharide (LPS; Sigma, USA) and 100 ng/mL interferon gamma (IFN-γ; R&D, USA) were added to induce the pro-inflammatory M1 phenotype. 60 ng/mL interleukin 4 (IL-4; Sigma, USA) was added to induce the anti-inflammatory M2 macrophages. The induction was conducted at 37 °C with 5% CO_2_ for 12 h. The cells were washed with phosphate buffered saline (PBS) for 3 times to completely remove the residue of LPS, IFN-γ, or IL-4. Phenotype-switched macrophages were starved for 24 h by incubated with equal volume of serum-free DMEM before conditioned medium collection (Huang et al. 2017). This conditioned medium derived from M1 and M2 macrophages, as well as the non-polarized group M0 were then collected for further experiments. The macrophage phenotype changes were further confirmed by measuring the content of M0, M1, and M2 macrophage-derived conditioned medium using ELISA kit (Proteome Profiler Mouse XL Cytokine Array, R&D Systems, ARY028).

### IDG-SW3 cell culture

IDG-SW3 (murine immortal osteocyte, EKC001, Kerafast, USA) cells were allowed to proliferate at 33 °C with α-MEM medium (Life Technologies, USA) containing 10 ng/mL IFN-γ at an initial seeding density of 5000 cells/cm^2^ on type I collagen-coated plates (0.2 mg/mL in 0.2 M acetic acid). The specific cell culture medium developed for the experiment was made by mixing the conditioned medium derived from M0, M1, M2 macrophages (named Group M0-CM, M1-CM, and M2-CM), respectively, with α-MEM medium (Life Technologies, USA) at a ratio of 1:1, supplemented with 50 µg/mL ascorbic acid (Sigma, USA) and 4 mM β-glycerophosphate (Sigma, USA) for 14 days of osteogenic differentiation. The mixed medium for osteocyte culturing was replaced every 3 days.

### Rat OA model

All animal experiment and protocols used in this project were approved by the Queensland University of Technology Animal Ethics Committee (Ethics No. 1400000274). Experimental OA group was induced in five 8-week-old female Wistar rats by surgically removal of meniscus of the right knee (Zhang et al. 2016a). The first step was transecting the medial collateral ligament of right knee just below its attachment to the meniscus. Then the narrowest point of meniscus was cut to letting complete medial meniscus transection. Sham operation on five 8-week-old female Wistar rats were regarded as a control. Rats were randomly divided into the sham group and OA group. All rats were sacrificed, and knee joints were collected 8 weeks post OA surgery. The rat knees were prepared for histological and immunohistochemical analysis.

### Scanning electron microscope (SEM)

SEM was used to examine the morphological change of osteocytes (Moran and Coats 2012). IDG-SW3 cell samples were cultured with macrophage-derived conditioned medium for 14 days. The cells were washed with PBS and then fixed with 2.5% glutaraldehyde (Sigma, G5882), followed by postfixation in 1% osmium tetroxide. The fixed cells were then processed through dehydration by a series of increasing ethanol concentrations (50 to 100% vol/vol), and gold-coated for observation of the cell morphology using SEM (FESEM, Zeiss Sigma) under 5kv using SE2 mode. For morphology calculation, 20 cells of each sample were randomly chosen, and the average cell length and cell width measured. Shape factor was used to quantify how rounded the osteocytes were. The shape factor was calculated using equation: S = 4πA/P^2^, A representing area of the cell, and P representing cell perimeter (Friel et al. 2000). The morphology and density distribution of mineralized collagen were captured with density-dependent color scanning electron micrographs (DDC-SEM) (Zhou et al. 2019; Bertazzo et al. 2013). DDC-SEM were obtained by merging images of the same collagen fiber region captured using both inlens mode and backscatter mode. Both images were stacked using ImageJ software, and the inlens image was assigned to the green channel whereas the backscatter image was assigned to the red channel. The ratio of calcium and phosphorus of each sample was detected by EDS under 20 kv. Twenty areas of mineralized collagens from each sample were randomly selected for EDS detection.

The non-decalcified normal and OA bone samples were fixed overnight in 4% paraformaldehyde (PFA). The specimens were dehydrated in a series of graded alcohol from 70 to 100%, 3 days for each step. The samples were then embedded in Technovit 7200 resin, sectioned into half using a diamond saw (Exakt Technologies, USA) and ground with 1200 grit sandpaper. The specimens were polished with 1 μm and 0.3 μm Alpha Micropolish Alumina II (Buehler) in a soft cloth rotating wheel. The surface of the resin-embedded samples was acid etched with 40% phosphoric acid for 15 s. The samples were then immersed in 5% sodium hypochlorite for 5 min. The acid etched specimens were washed with distilled water, left to air dry, and then coated with 2 nm of gold. The SE2 detection process was conducted under voltage 5 kv. Twenty cells in the bone remodeling area of each sample were randomly chosen for morphology calculation. The cell shape factor was calculated as described above. The backscattered scanning electron microscope (BSEM) can detect density distribution for bone samples so it was applied here (Marshall et al. 2003). The non-acid etched bone samples were examined with SEM (FESEM, Zeiss Sigma) under voltage 20 kv with BSEM mode. The ratio of calcium and phosphorus of each sample was detected by EDS in the same condition. Twenty areas of each sample were randomly selected for EDS detection.

### Transmission electron microscope (TEM)

TEM was applied to generate high resolution images of ultra-thin specimens (Keene and Tufa 2010) for the observation of the combined collagen and mineral-crystal particles. Preparation of the macrophage-derived conditioned medium stimulated cell samples were performed followed the fixation and dehydration process for TEM samples. Mineralized cultures were fixed in 2.5% glutaraldehyde at 4 °C for 1 h, postfixed in 1% osmium tetroxide, dehydrated in a graded ethanol series, treated with acetonitrile, and finally infiltrated with a LE2 resin. Embedded samples were polymerized at 60 °C for 24 h, sectioned (75 nm) using Leica EM UC7 ultramicrotome and collected on bare 300 mesh copper TEM grids followed by post-staining with uranyl acetate and lead citrate. TEM observation was performed using JEM-1400 TEM (JEOL, Japan) at 80 kV. Selected-area electron diffraction (SAED) was used to distinguish the orientation of mineralized collagen, which presents the arrangement of hydroxyapatite (HA) in bone samples (Fujisaki and Tadano 2007). Selected-area aperture for the diffraction observation of the samples was a circular area of 100 nm in diameter with an acceleration voltage of 100 kV.

### Green fluorescence detection

Mature IDG-SW3 cells express DMP1-GFP, at which stage green fluorescence can be detected (Woo et al. 2011). IDG-SW3 cells were cultured with M1, M2, or non-polarized M0 macrophage derived conditioned medium for 14 days, as detailed above. The live cells were imaged with NIKON ECLIPSE Ti microscope at time point day 7 and day 14. The nuclei were visualized using Hoechst 33342 (Thermo Fisher Scientific, USA). The fluorescence intensity was quantified using ImageJ software and presented with intensity arbitrary unit (AU).

### Gene expression detection

Total RNA of osteocyte samples from Group M0-CM, M1-CM, and M2-CM were extracted after 1, 3, 7, and 14 days of culture, using TRIzol® reagent (Life Technologies Pty Ltd., Australia) (Shao et al. 2018a). The purity and quantity of RNA was determined spectrophotometrically using a NanoDrop instrument (Thermo Fisher Scientific Inc.). The cDNA was synthesized from 1 μg of total RNA using the SensiFAST™ cDNA Synthesis Kit (Bioline Australia Pty Ltd.). The real-time quantitative reverse transcription polymerase chain reaction (qRT-PCR) was performed using a QuantStudio 7 Flex Real-Time PCR System (Applied Biosystems, Thermo Fisher Scientific) with SYBR Green reagent to detect the expression of osteocyte markers (*E11* and *Dmp1*) and Notch signaling pathway marker (*Hes1*). Relative gene expression was normalized against *Gapdh* and calculated as previously described (Livak and Schmittgen 2001). The primers used in this part of the study are listed below (Table 1). All experiments were performed in triplicate for each condition and repeated three times.

**Table 1:** Primers used for qRT-PCR

Target gene	Forward primer	Reverse primer
*E11*	5′-aaacgcagacaacagataagaaagat-3′	5′-gttctgtttaggtctttagggcga-5′
*Dmp1*	5′-agatccctcttcgagaacttcgct-3′	5′-ttctgatgactcactgttcgtgggtg-3′
*Hes1*	5′-cagctgacaaggaggactga-3′	5′-gtcacctcgttcatgcactc-3′
*Gapdh*	5′-gtgtccgtcgtggatctga-3′	5′-cctgcttcaccaccttcttg-3′

### Alizarin Red S staining

Cell samples were collected after 14 days cultured with macrophage-derived conditioned medium and fixed with 4% PFA. The fixed cell samples were incubated with 1% Alizarin Red S (A5533, Sigma-Aldrich) for 20 min and washed with distilled water. Alizarin Red S of each sample was extracted with 300 μL 50% acetic acid, and then incubated for 30 min at room temperature. The dye solutions were neutralized with 10% ammonium hydroxide (320145, Sigma-Aldrich), and the pH adjusted to 4.1. The solutions were centrifuged at 10,000 RPM for 10 min. 100 μL supernatant of each sample was transferred into 96-well plates and detected by BIO-RAD microplate absorbance spectrophotometer at 405 nm.

### Tissue preparation

The sham operated and OA rat joints containing subchondral bone were isolated and then fixed in 4% PFA overnight (Sun et al. 2017). The samples were then decalcified in 10% ethylenediaminetetraacetic acid (EDTA, pH 7.1), and embedded in paraffin wax after the decalcification was complete. The paraffin blocks were sectioned using microtome to 5 μm for histological and immunohistochemical analysis.

### Hematoxylin and eosin (H&E), Safranin O, and tartrate-resistant acid phosphatase (TRAP) staining

The paraffin sections of bone samples were dewaxed in xylene and rehydrated in descending concentrations of ethanol from 100 to 70%. For H&E staining, the slides were stained with Mayer’s hematoxylin for 3 min, then washed with tap water for 5 min. The slides were dehydrated with ethanol from 70 to 100% and stained with eosin for 15 s. The slides were finally cleared with xylene and mounted on DePeX mounting medium (BDH Laboratory Supplies, England).

For Safranin O staining, the slides were incubated with Weigert’s iron hematoxylin after dehydration and washed with running tap water. The slides were then stained with Fast Green for 5 min, followed by a quick rinse with 1% acetic acid solution for 10 s. The slides were then stained in 0.1% Safranin O solution for 5 min, dehydrated with ethanol and stained with eosin for 15 s. The slides were cleared with xylene and mounted.

For TRAP staining, the slides were incubated for 20 min in acetate buffer prepared by 0.2 M sodium acetate (S2889, Sigma) and 50 mM tartaric acid (251380, Sigma) after dehydration. Then 0.5 mg/mL naphthol AS-MX phosphate (N4875, Sigma) and 1.1 mg/mL of Fast Red TR (F6760, Sigma) salt were added to the acetate buffer, and the sections were incubated for 4 h, followed by counterstaining with Mayer’s hematoxylin. Images of H&E, Safranin O, and TRAP-stained slides were captured using Axion software under the light microscope (Carl Zeiss Microimaging).

### Immunohistochemical staining

Following the dewaxing, hydration and antigen retrieval, the slides were incubated with 3% H_2_O_2_ for 20 min to quench endogenous peroxidase enzymes and 1% bovine serum albumin (BSA, A3608, Sigma) blocking buffer for 1 h to reduce nonspecific binding. Primary antibodies against CD68 (ab125212, Abcam), iNOS (PA1-036, Thermo Fisher Scientific), CD206 (ab64693, Abcam), Podoplanin (E11) (ab10288, Abcam), DMP1 (ab103203, Abcam), HES1 (ab108937, Abcam) were applied at 1:100 dilutions and incubated at 37 °C for 2 h, and rabbit immunoglobulin G (IgG, ab171870, Abcam) was regarded as primary antibody for isotype control. This process was followed by incubation with biotinylated swine-anti-mouse, rabbit, goat secondary antibody (DAKO) for 45 min, and diaminobenzidine (DAB) solution (DAKO) was then applied to visualize the protein-antibody complex. The slides counterstained with Mayer's hematoxylin. Images of all slides were captured using Axion software under the light microscope (Carl Zeiss Microimaging). The immunohistochemistry (IHC) images were quantified using ImageJ software by calculating the percentage of positive cells per field of view (FOV), and 5 FOVs of each sample were calculated.

### Immunofluorescence staining

After dewaxing and hydration, tissues on slides were treated with proteinase K for 20 min for antigen retrieval. The endogenous peroxidase activity was eliminated by incubating the sections in 3% H_2_O_2_ for 20 min. Non-specific protein binding was blocked with 1% BSA (A3608, Sigma) for 1 h. Slides were then incubated with primary antibodies overnight at 4 °C. Antibodies for macrophage specific proteins were rabbit anti-CD68 (ab125212, Abcam) and rat anti-CD11b (ab64347, Abcam) as macrophage pan markers, mouse anti-CD86 (sc-28347, Santa Cruz Biotechnology) and rabbit anti-iNOS (PA1-036, Thermo Fisher Scientific) as M1 macrophage markers, and rabbit anti-CD206 (ab64693, Abcam) and mouse anti-CD163 (ab156769, Abcam) as M2 macrophage markers. For the osteocyte specific markers, antibodies used were mouse anti-Podoplanin (E11, ab10288, Abcam) and rabbit anti-DMP1 (ab103203, Abcam). For the marker of Notch signaling pathway, the antibody used was anti-HES1 (ab108937, Abcam). After primary antibody incubation, secondary antibodies were added on slides and incubated at 4 °C overnight. The secondary antibodies applied to slides were goat anti-mouse IgG Alexa Fluor 488 (A-11001, Life Technologies) and Alexa Fluor 568 (A-11004, Life Technologies), goat anti-rabbit IgG Alexa Fluor 488 (A-11034, Life Technologies) and Alexa Fluor 568 (A-11036, Life Technologies), and goat anti-rat Alexa Fluor 488 (A-11006, Life Technologies) and Alexa Fluor 568 (A-11077, Life Technologies). Unbonded secondary antibodies were removed by washing the slides using PBS. The slides were then counterstained with DAPI (D1306, Life Technologies) and mounted with ProLong® Gold Antifade Reagent (P10144, Life Technologies). Images of immunofluorescence (IF)-stained slides were observed and captured using Nikon A1R confocal microscope.

### Western blot

The protein of each cell sample was collected and suspended in 500 μL RIPA lysis buffer (R0278, Sigma) with protease inhibitor (cOmplete, EDTA-free 04693132001, Roche) and phosphatase inhibitor (PhosSTOP, 04906845001, Roche). A total of 20 μg of proteins from each sample were loaded and separated on SDS-PAGE gels. The separated proteins were transferred onto a nitrocellulose membrane (Pall Corporation) at 4 °C by a constant current of 10 mA. Odyssey blocking buffer (P/N 927–40,000, LI-COR Biosciences) was added to protein-contained membranes to block the unspecific binding. The membranes were incubated with primary antibodies HES1 (1:1000, ab108937, Abcam), E11 (1:1000, ab10288, Abcam), DMP1 (1:1000, ab103203, Abcam), and α-Tubulin (1:2000, ab15246, Abcam) overnight at 4 °C. Unbonded primary antibodies were removed by washing twice in PBS containing 1% Tween 20 (Bio-Rad, USA). The membranes were then incubated with anti-mouse/rabbit fluorescence conjugated secondary antibodies at 1:10,000 dilutions for 1 h at room temperature. Unbonded secondary antibodies were removed by washing twice in PBS containing 1% Tween 20. The protein bands were visualized using the Odyssey Infrared Imaging System (LI-COR Biosciences). The relative intensity of protein bands was quantified using ImageJ software.

### Activation of Notch signaling pathway

Artificial activation of Notch by Notch (8G10) antibody was established and reported by Conboy et al. (2003), and has been proven for successful Notch activation in osteocytes (Shao et al. 2018b). Culture plates were coated with collagen I and then coated with anti-Notch 1 antibody (extracellular, clone 8G10, Cat#MAB5414, Merck Millipore) at 1:100 dilution in PBS at 4 °C overnight. The plates of control group were coated with goat IgG at 1:100 dilution in PBS. The successfully activation of Notch signaling pathway in osteocytes was confirmed by western blot analysis.

### Statistical analysis

Data were expressed as mean ± standard deviation (SD) for three independent experiments performed under the same condition. Statistical differences between each group were determined by one-way ANOVA with Bonferroni’s multiple comparison tests (assuming equal variances), and Student’s t-test was applied to determine the statistical differences between the sham and OA groups. Because cells from each group were cultured under the same condition, multiple linear regression analyses and analysis of covariance were applied to test for the differences of morphological factors and genes between groups at each specific time point. Similar analysis was performed to test for the differences of the sham and OA groups as rats of the same age/gender were fed under the same condition. One-way ANOVA test and Student’s t-test were performed with GraphPad Prism 9 software. A *p* < 0.05 was considered statistically significant.

## Results

### M1 macrophages caused morphology changes on osteocytes

**Fig. 1:**
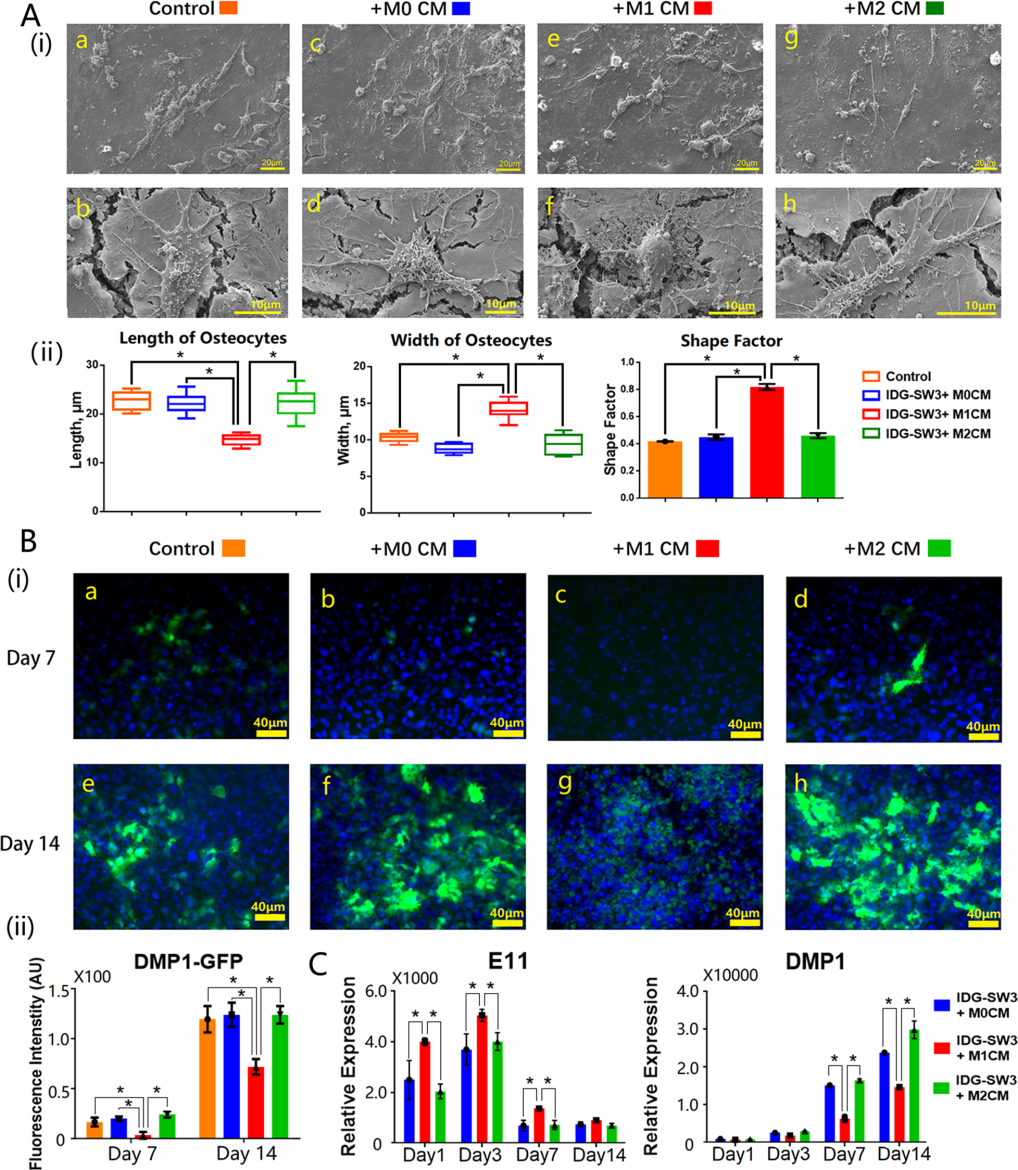
M1 macrophages inhibited the maturation of osteocytes. **A**(i) Representative SEM images of osteocytes cultured with macrophage-derived conditioned medium (a, c, e, g: low magnification at ×500 and scale bars represented 20 μm; b, d, f, h: high magnification at ×5000 and scale bars represented 10 μm); **A**(ii) The box plot and bar graphs showed the morphology changes of osteocytes after 14 days simulation with macrophage-derived conditioned medium, 20 randomly selected areas from each sample were analyzed, and data shown as the mean ± SD (*p < 0.05, one-way ANOVA); **B**(i) fluorescence images of macrophage-derived conditioned medium-treated osteocytes (scale bars represented 40 μm); **B**(ii) the intensity of DMP-GFP was measured using ImageJ software; **C** gene expression of osteocyte markers after 14 days simulation with macrophage-derived conditioned medium, and data from three independent experiments performed under the same condition were shown as mean ± SD (*p < 0.05, one-way ANOVA)

Representative SEM images showed that the morphological changes of osteocytes cultured with macrophage-derived conditioned medium (CM) (Fig. 1A). By observing and analyzing 20 randomly selected areas from M0-CM, M1-CM, M2-CM, and untreated group, respectively, it was obvious that the overall shape of osteocytes cultured with M1-CM (Fig. 1A(i)—e, f) were different from the M0-CM (Fig. 1A(i)—c, d) and M2-CM treated osteocytes (Fig. 1A(i)—g, h). Both osteocytes cultured with M0-CM and M2-CM presented in a spindle-like shape of 20 μm in length and 10 μm in width (Fig. 1A(ii)). The shape factor of osteocytes in various treatment groups were around 0.45, however, significantly increased shape factor up to 0.82 ± 0.02 was found on M1-CM treated osteocytes (Fig. 1A(ii)), reflecting cell shape transformation from being spindle-like to round shape after being cultured with M1-CM.

### M1-stiumulated osteocytes were unable to reach maturity

IDG-SW3 osteocyte cell line has a GFP gene attached at the end of DMP1 gene, which means the expression of DMP1 protein will show a green fluorescence (Woo et al. 2011). The Epi-fluorescence images were obtained from osteocytes cultured with macrophage-derived CM for 7 and 14 days (Fig. 1B(i)). It can be identified that M1-CM treated group expressed the least DMP1 (Fig. 1B(ii)), which presented the least green fluorescence throughout day 7 and day 14 (Fig. 1B(i)—c, g). To detect the effect of macrophages on osteocyte maturation, qRT-PCR was used to measure the gene expression of early and late osteocyte markers on day 1, 3, 7, and 14 (Fig. 1C). The results show osteocyte mature marker DMP1 decreased from day 7 to 14 when osteocytes were cultured with M1-CM compared to M0-CM and M2-CM. However, M1-CM treated osteocytes showed an increased expression level of osteocyte early marker E11, compared to M0-CM and M2-CM which remained until day 7. The protein expression level of M0-CM, M1-CM, and M2-CM detected via western blot further confirmed the increased E11 expression and downregulated DMP1 expression in M1 macrophage-stimulated osteocytes (Additional file 1: Fig. S1B).

### M1 macrophages affected mineralized nodule formation in osteocyte cultures

**Fig. 2:**
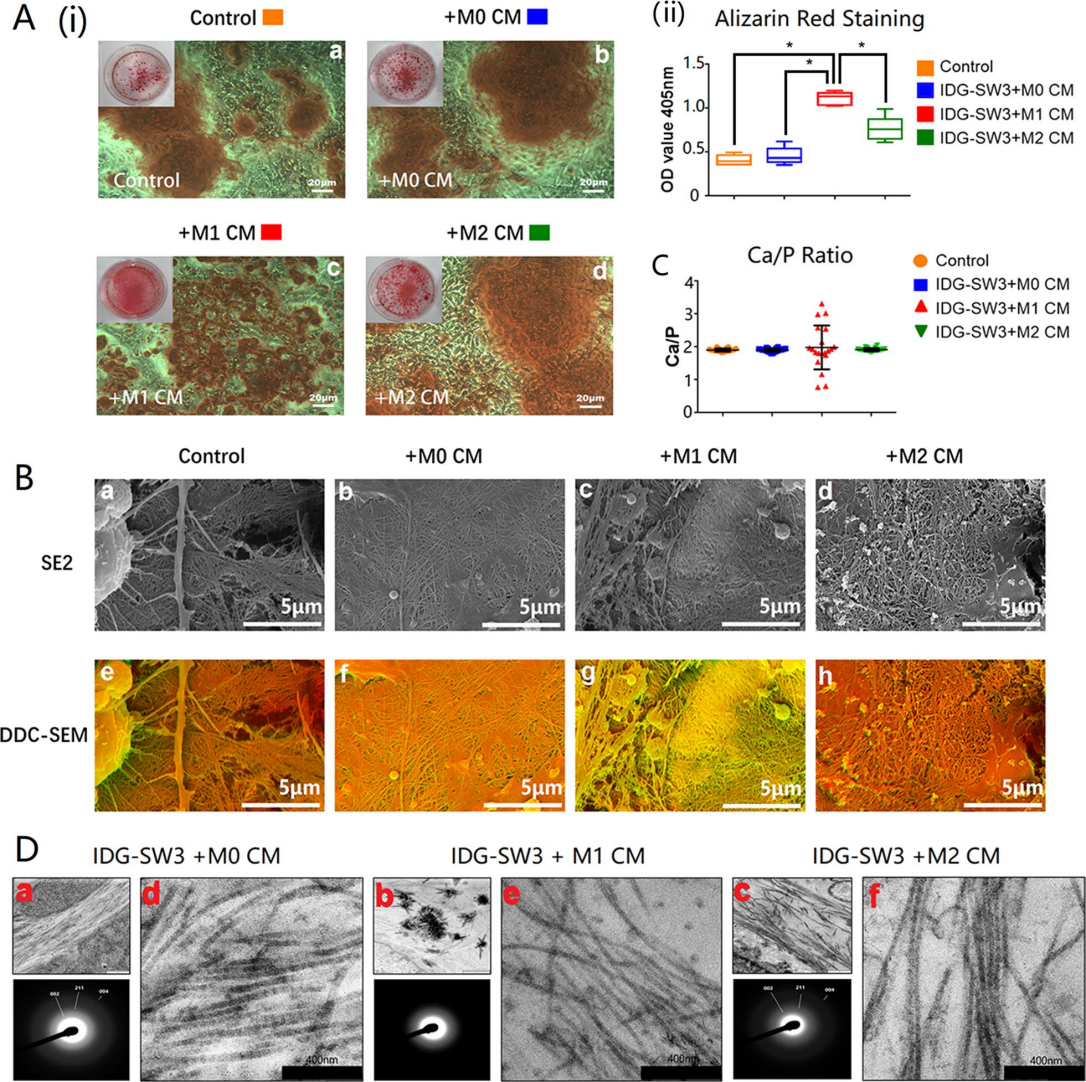
M1 macrophages negatively affected osteocyte mineralization. **A** Alizarin Red S staining images of osteocytes cultured with macrophage-derived conditioned medium. The box plot graph showed the average optical density (OD) value of Alizarin Red S staining and the OD values were shown as the mean ± SD (*p < 0.05, one-way ANOVA); **B** SEM images of mineralized collagen fibers synthesized from macrophage-derived conditioned medium treated osteocytes. Images captured by SE2 mode were displayed above and DDC-SEM images were displayed underneath the SE2 images (scale bars represented 5 μm); **C** Ca/P ratio of mineralized collagen was detected with EDS by randomly measuring 20 points from each sample; **D** TEM images and X-ray diffraction patterns demonstrated the ultrastructure of mineralized collagen synthesized by macrophage-stimulated osteocytes

Alizarin Red S positive staining appeared on matrix after 14 days culturing osteocytes with the macrophage-derived CM. The images of Alizarin Red S-stained osteocytes showed that osteocytes cultured with M1 macrophage CM (M1-CM) had a greater amount of mineralized nodule formation (Fig. 2A(i)), which was further proven by presenting the highest intensity among all samples detected using the microplate absorbance spectrophotometer (Fig. 2A(ii)). However, when observed under high magnification, the morphology of mineralized nodules formed from M1-CM treated osteocytes (Fig. 2A(i)—c) were smaller, and their distribution scattered, compared with those from M0-CM (Fig. 2A(i)—b) and M2-CM (Fig. 2A(i)—d) treated ones, or untreated osteocytes (Fig. 2A(i)—a).

### M1 macrophages negatively affected the mineralization of collagen fibers

DDC-SEM images could help to demonstrate the density distribution of mineralized collagen fibers, in which high density areas shown in red and low-density areas shown in green. The DDC-SEM images identify that the density distribution of mineralized collagen from M1-CM stimulated osteocytes was lower and uneven compared with those from M0-CM, M2-CM, or untreated samples (Fig. 2B). The average Ca/P ratios were similar around 1.90 in samples treated with M0-CM and M2-CM, as well as the untreated samples, while the Ca/P ratio mean value of M1-CM treated mineralized collagen fluctuated, indicating the altered mineralization with various Ca/P ratios (Fig. 2C).

### M1 macrophages negatively affected the ultrastructure of mineralized collagen fibers

TEM was applied to observe the ultrastructure of mineralized collagen from macrophage-derived CM-stimulated osteocytes (Fig. 2D). Black-white bands, revealing the combination of minerals into collagen gap zones, did not appear in M1-CM treated osteocytes (Fig. 2D(b, e)). Contrastingly, spontaneously aggregated minerals were found in M1-CM treated samples (Fig. 2D(b)). Mineralized collagen synthesized from M0-CM or M2-CM treated osteocytes characterized by sharp and distinct diffraction rings, namely 002, 004, and 211 (Fig. 2D(d, f)). The diffraction pattern was less distinct in mineralized collagen synthesized by M1-CM treated osteocytes (Fig. 2D(e)).

### Osteocyte morphology changed in inflammatory bone remodeling areas

**Fig. 3:**
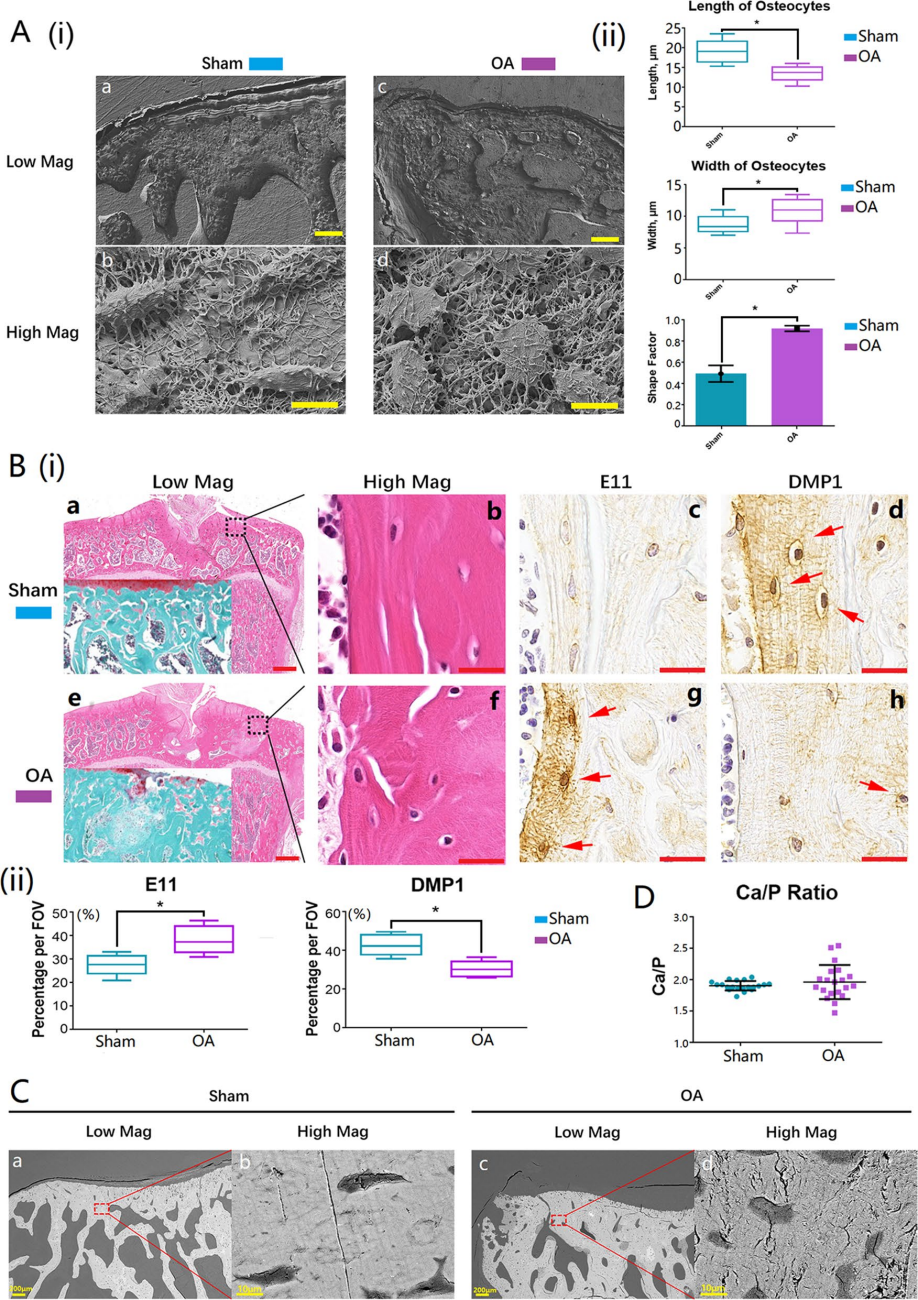
Osteocytes were immature and mineralization was abnormal in inflammatory bone remodeling areas in OA samples. **A**(i) Representative SEM morphological images of osteocytes in normal and OA inflammatory bone remodeling areas (a and c: scale bars represented 100 μm in low magnification; b and d: scale bars represented 10 μm in high magnification); **A**(ii) the box plot and bar graphs showed the morphology changes of osteocytes in normal and OA samples, 20 randomly selected cells from each sample were analyzed, and data shown as the mean ± SD (*p < 0.05, t-test); **B**(i) H&E, Safranin O, and IHC staining images of normal and OA inflammatory bone remodeling areas (E11: early osteocyte marker; DMP1: mature osteocyte marker; red arrows showed the positive cells; a and e: scale bars represented 500 μm in low magnification; b, c, d, f, g, and h: scale bars represented 20 μm in high magnification); **B**(ii) the box plot graphs showed the percentage of the positive cells per field of view (*p < 0.05, t-test); **C** BSEM images of subchondral bone remodeling areas from normal and OA bones (the magnification was ×100; scale bars represented 200 μm); **D** Ca/P ratios of bone remodeling areas from normal and OA subchondral bones were detected with EDS, and 20 randomly chosen points were measured

The morphology differences of osteocytes from bone remodeling areas of normal and OA subchondral bone were obvious (Fig. 3A(i)). In the normal samples, the average length and average width of osteocytes was 19.1 ± 3.1 μm and 8.2 ± 2.2 μm (Fig. 3A(ii)), while in the OA samples, the average length and average width of osteocytes was 13.3 ± 2.3 μm and 10.7 ± 2.2 μm (Fig. 3A(ii)). Similar to M1 macrophage-stimulated osteocytes, the general morphology of osteocytes in the OA bone remodeling areas were round (Fig. 3A(i)—c, d), whereas the cells were spindle-shape in the normal bone remodeling areas (Fig. 3A(i)—a, b), which was evidenced by a high shape factor of up to 0.90 ± 0.03 in OA samples (Fig. 3A(ii)).

### Osteocyte maturation was inhibited in inflammatory bone remodeling areas

Images of H&E staining identified the areas of interest in the bone samples (Fig. 3B(i)—b, f). The represented images of Safranin-O staining showed slight damage on cartilage on OA section under which the subchondral bone was exposed (Fig. 3B(i)—a, e). The selected osteocyte-specific proteins were E11 as the immature osteocyte marker and DMP1 as the mature osteocyte marker (Fig. 3B(i), Additional file 2: Fig. S2c, d, g, and h) (Zhang et al. 2006; Rios et al. 2005; Feng et al. 2006). Similar to what was found in the M1 macrophage-stimulated osteocytes in vitro, the IHC images demonstrate that the osteocytes in OA sections expressed a higher level of E11 but a lower level of DMP1 (Fig. 3B(i)—g, h), while the opposite occurred in the sham operated sections (Fig. 3B(i)—c, d; Fig. 3B(ii)).

### Mineralization was abnormal in inflammatory bone remodeling areas

The density distribution of bone samples can be presented using the backscattered SEM (BSEM) mode (Marshall et al. 2003). The BSEM images of normal and OA bone samples from rat knees showed obvious differences in density distribution. Density in the normal bone samples was evenly distributed (Fig. 3C(a, b)), while it was uneven in the OA samples (Fig. 3C(c, d)). The darker color shown in the bone remodeling areas in the OA subchondral bones demonstrated that the density of bone mineralization in the OA inflammatory bone remodeling areas was lower than that in normal bones (Fig. 3C). The ratio of calcium and phosphorus was detected using the EDS detector. The average Ca/P ratios were around 1.94 in both normal and OA bones, however the value of Ca/P in the OA bones varied compared with the normal bones (Fig. 3D).

### Involvement of macrophage polarization in inflammatory bone remodeling

**Fig. 4:**
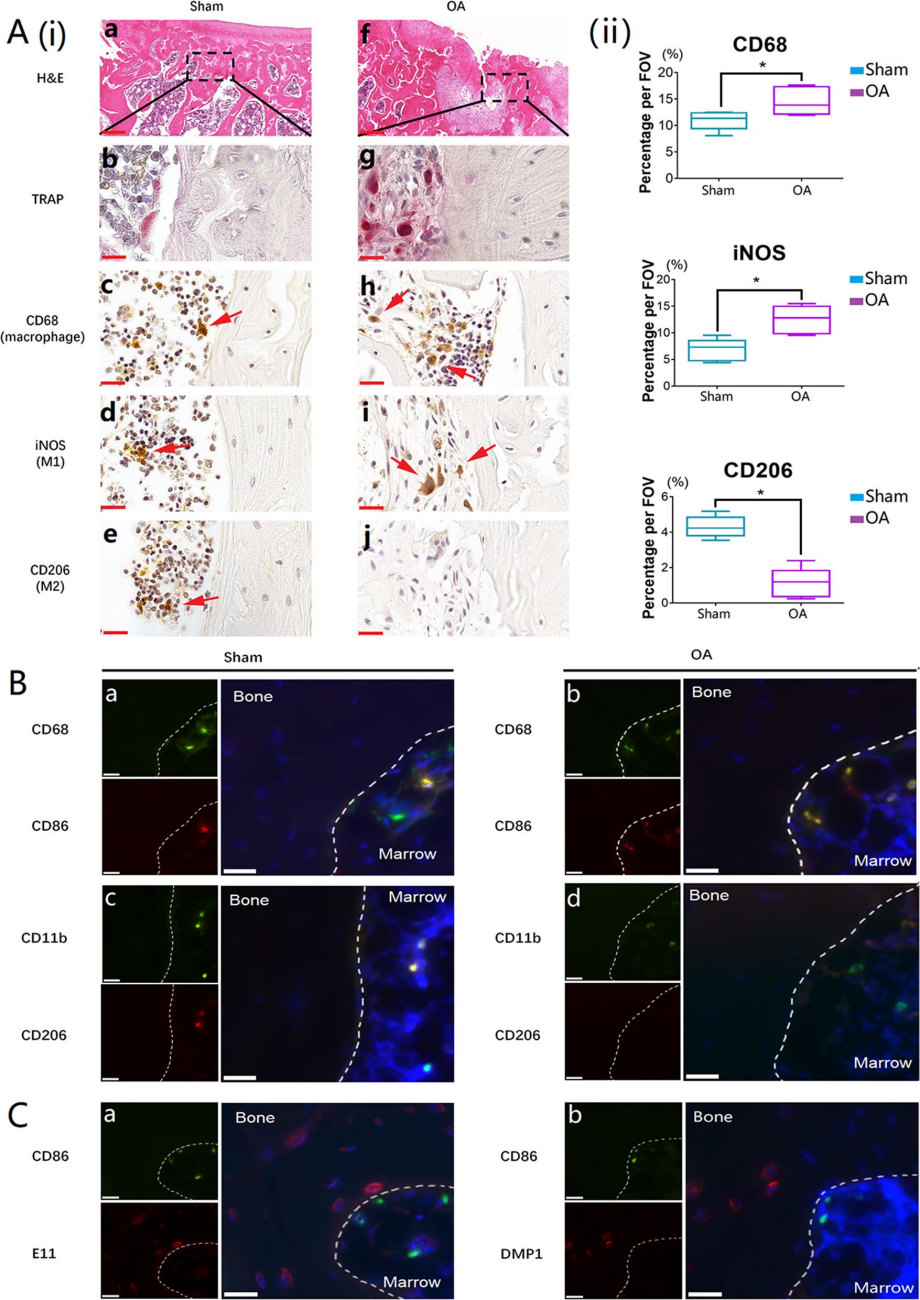
Macrophages were actively involved in inflammatory bone remodeling and M1 macrophages were the major activated macrophage subtype in OA bone remodeling areas. **A**(i) H&E, TRAP, and IHC staining of macrophage specific markers (CD68: macrophage pan marker; iNOS: M1 macrophage marker; CD206: M2 macrophage marker; positive cells were labeled with red arrows) on normal and OA bone sections (a and f: scale bars represented was 200 μm in H&E staining; b and g: scale bars represented was 20 μm in TRAP staining; c, d, e, h, i, and j: scale bars represented was 20 μm in IHC staining); A(ii): The box plot graphs demonstrated the positive cells per field of view. Data was shown as the mean ± SD (*p < 0.05, t-test); **B** IF double staining of macrophage markers on normal and OA bone sections (CD68 and CD11b: macrophage pan markers; CD86: M1 macrophage marker, CD206: M2 macrophage marker; scale bars represented 20 μm); **C** IF double staining of M1 macrophage marker and osteocyte marker on OA bone sections (CD86: M1 macrophage marker; E11: early osteocyte marker; DMP1: mature osteocyte marker; scale bars represented 20 μm)

H&E staining and TRAP staining clearly showed that bone remodeling was dynamic in subchondral bone under defective cartilage (Fig. 4A(i)—a, b, f, and g). CD68 and CD11b are pan markers for the macrophage lineage, iNOS is regarded as the marker for M1 phenotype, whereas CD206 is that for M2 phenotype (Huang et al. 2017). Representative IHC images showed the percentage of CD68 positive cells in the OA samples is significantly higher than that in the normal samples (Fig. 4A(i)—c, h; Fig. 4A(ii)). The percentage of iNOS positive cells in OA bones was higher than that in normal bones (Fig. 4A(i)—d, i; Fig. 4A(ii)). On the contrary, less CD206-expressed cells were present in OA bones compared with normal bones (Fig. 4A(i)—e, j; Fig. 4A(ii)). The representative IF images readily identified that almost all the CD68 positive cells were CD86 positive in the OA inflammatory bone remodeling areas (Fig. 4B(c)), whereas nearly no CD11b/CD206 positive cells were found (Fig. 4B(d)). The phenotype of macrophages involved in inflammatory bone remodeling areas were further proven by IF double staining with M1 markers (CD86/iNOS, Additional file 3: Fig. S3a, b) and M2 markers (CD206/CD163, Additional file 3: Fig. S3c, d). In OA inflammatory bone remodeling areas, representative IF images showed that E11 positive osteocytes appeared to be adjacent to CD86 positive cells (M1 macrophages) (Fig. 4C(a)), while DMP1 positive osteocytes identified far away from CD86^+^ M1 macrophages (Fig. 4C(b)).

### M1 macrophage-derived CM inhibited the activation of Notch signaling in vitro

**Fig. 5:**
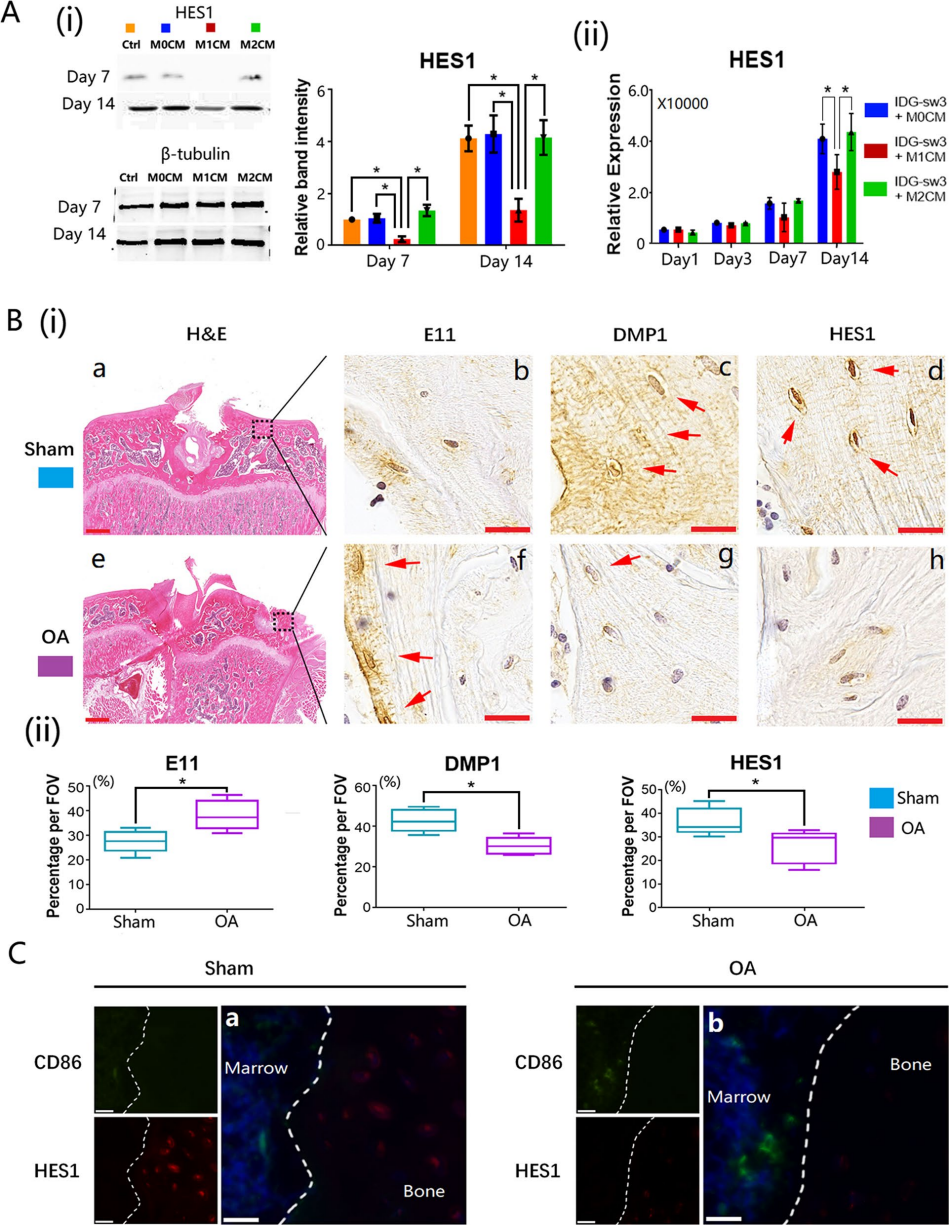
Notch signaling pathway was inhibited in M1 macrophage-stimulated osteocytes and Notch signaling pathway was downregulated in OA inflammatory bone remodeling areas. **A**(i) Expression of HES1 from macrophage-derived medium cultured osteocytes detected by western blot; **A**(ii) qRT-PCR measurement of HES1 gene expression from macrophage-derived medium cultured osteocytes. Data from three independent experiments performed under the same condition were shown as mean ± SD (*p < 0.05, one-way ANOVA); **B** normal and OA bone sections stained with H&E and IHC (E11: immature osteocyte marker; DMP1: mature osteocyte marker; HES1: Notch signaling pathway marker; a and e: scale bar present was 100 μm; b, c, d, f, g, and h: scale bar present was 20 μm); **C** IF double staining images of normal and OA bone sections (CD86: M1 macrophage marker; HES1: Notch signaling pathway marker; the scale bars present was 20 μm)

The expression level of HES1 can reflect the status of the Notch signaling pathway (Zanotti et al. 2011). The protein expression level was significantly lower in M1 macrophage-stimulated osteocytes than in M0 macrophage- or M2 macrophage-treated osteocytes (Fig. 5A(i)). These lower HES1 expression levels were consistent from day 7 until day 14, as shown in the western blot results (Fig. 5A(i)). It was also confirmed in qRT-PCR detection that, the gene expression of HES1 in M1 macrophage-stimulated osteocytes was downregulated throughout 14 days of co-culture (Fig. 5A(ii)) which means that the Notch signaling pathway was inhibited in osteocytes co-cultured with the M1 macrophage-derived CM.

### Notch signaling activation was inhibited in inflammatory bone remodeling areas

Representative IHC staining images showed that the expression of HES1 in osteocytes from OA bones was downregulated compared with normal bones (Fig. 5B(i)—d, h; Fig. 5B(ii)). The downregulation of Notch signaling pathway was accompanied with a higher early osteocyte marker E11 and lower mature marker DMP1 in OA osteocytes (Fig. 5B(i)—b, f, c, and g; Fig. 5B(ii)). Linking to the phenomenon found in Fig. 3, both osteocyte maturation and the Notch signaling pathway were inhibited in OA inflammatory bone remodeling areas. Double IF staining using HES1 and M1 macrophage marker (CD86) were conducted in normal and OA bone sections with the normal bone sections showing fewer M1 macrophages while the Notch was highly activated in osteocytes (Fig. 5C(a)). However, in the OA bone sections, in which M1 macrophages were the major macrophage subtype, the Notch was inhibited in osteocytes (Fig. 5C(b)). To further investigate the role of Notch signaling pathway in inflammatory mineralization, Notch signaling was activated in osteocytes stimulated with M1-CM by culturing the cells on anti-Notch 1 antibody-coated plate. Western blot and qRT-PCR analysis on day 1, 3, 7, and 14 showed an upregulated expression of HES1 on both protein and gene levels, indicating that the Notch signaling pathway was successfully induced in M1 macrophage-treated osteocytes (Additional file 4: Fig. S4).

### Activation of Notch signaling pathway reversed the negative effects of M1 macrophages on osteocyte morphology

**Fig. 6:**
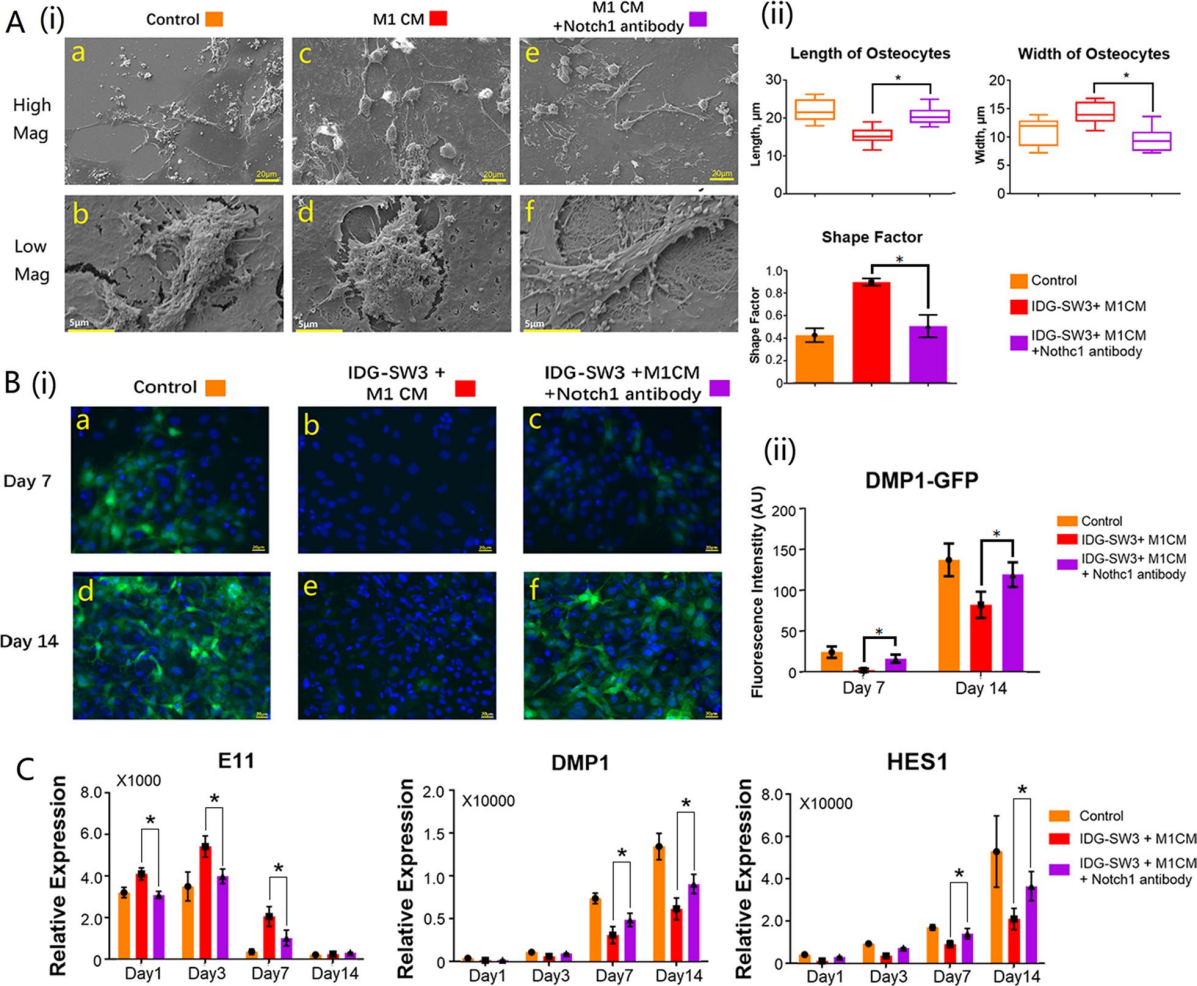
Activation of Notch reversed the negative effects of M1 macrophages on osteocyte morphology and maturation. **A**(i) SEM images showed osteocyte morphology changed after activation of Notch signaling pathway (a, c and e: Low magnification at ×500 and the scale bars represented 20 μm; b, d and f: High magnification at ×5000 and the scale bars represented 5 μm); **A**(ii) The bar graphs showed the morphology changes of osteocytes after 14 days simulation with macrophage-derived conditioned medium with or without activation of Notch signaling pathway. A total of 20 randomly selected areas from each sample were analyzed, and data shown as the mean ± SD (*p < 0.05, one-way ANOVA); **B**(i) epi-fluorescence images of M1 macrophage-stimulated osteocytes after Notch activation; **B**(ii) the intensity of DMP-GFP was measured using ImageJ software; **C** gene expression of osteocyte markers after 14 days simulation with macrophage-derived conditioned medium, with or without activation of Notch signaling pathway. Data from three independent experiments performed under the same condition were shown as mean ± SD (*p < 0.05, one-way ANOVA)

SEM images captured under SE2 mode provided vision of morphological changes of osteocytes with or without Notch signaling activation. As demonstrated in this study, osteocytes presented with round shape under stimulation of M1 macrophages. The morphology of M1 macrophage-stimulated osteocytes changed after the activation of Notch signaling pathway. The average length and width of 20 randomly selected cells from M1 macrophage-stimulated osteocytes with or without Notch signaling activation were calculated (Fig. 6A(ii)). The shape factor of M1 stimulated-osteocytes dramatically dropped from 0.90 ± 0.03 to 0.51 ± 0.09 after Notch signaling activation (Fig. 6A(ii)), which indicates that the shape of M1 macrophage-stimulated osteocytes (Fig. 6A(i)—c, d) were no longer round after Notch activation (Fig. 6A(i)—e, f), and the cell morphology became comparable to normal mature osteocytes in the control group (Fig. 6A(i)—a, b).

### Activation of Notch signaling reversed the negative effects of M1 macrophages on osteocyte maturation

Our results indicate that osteocytes were unable to reach maturity following co-culture with the M1 macrophage-derived CM, expressing a low level of mature marker DMP1 throughout the 14 days co-culture (Fig. 6B(i)—b, e), whereas the osteocytes in the control group were already mature on day 14 (Fig. 6B(i)—a, d). Representative confocal images show that the green fluorescence could be detected in M1 macrophage-stimulated osteocytes from day 7 to day 14 after being Notch activated (Fig. 6B(i)—c, f; Fig. 6B(ii)) indicating that DMP1 expression in M1 macrophage-stimulated osteocytes was upregulated via the Notch activation. The Notch activation downregulated the early osteocyte marker E11 in M1 macrophage-stimulated osteocytes at early stage from day 1 to day 7, while it increased the expression of DMP1 in M1 macrophage-stimulated osteocytes at day 7 and day 14 (Fig. 6C).

### Activation of Notch signaling reversed the negative effects of M1 macrophages on the bone nodule formation

**Fig. 7:**
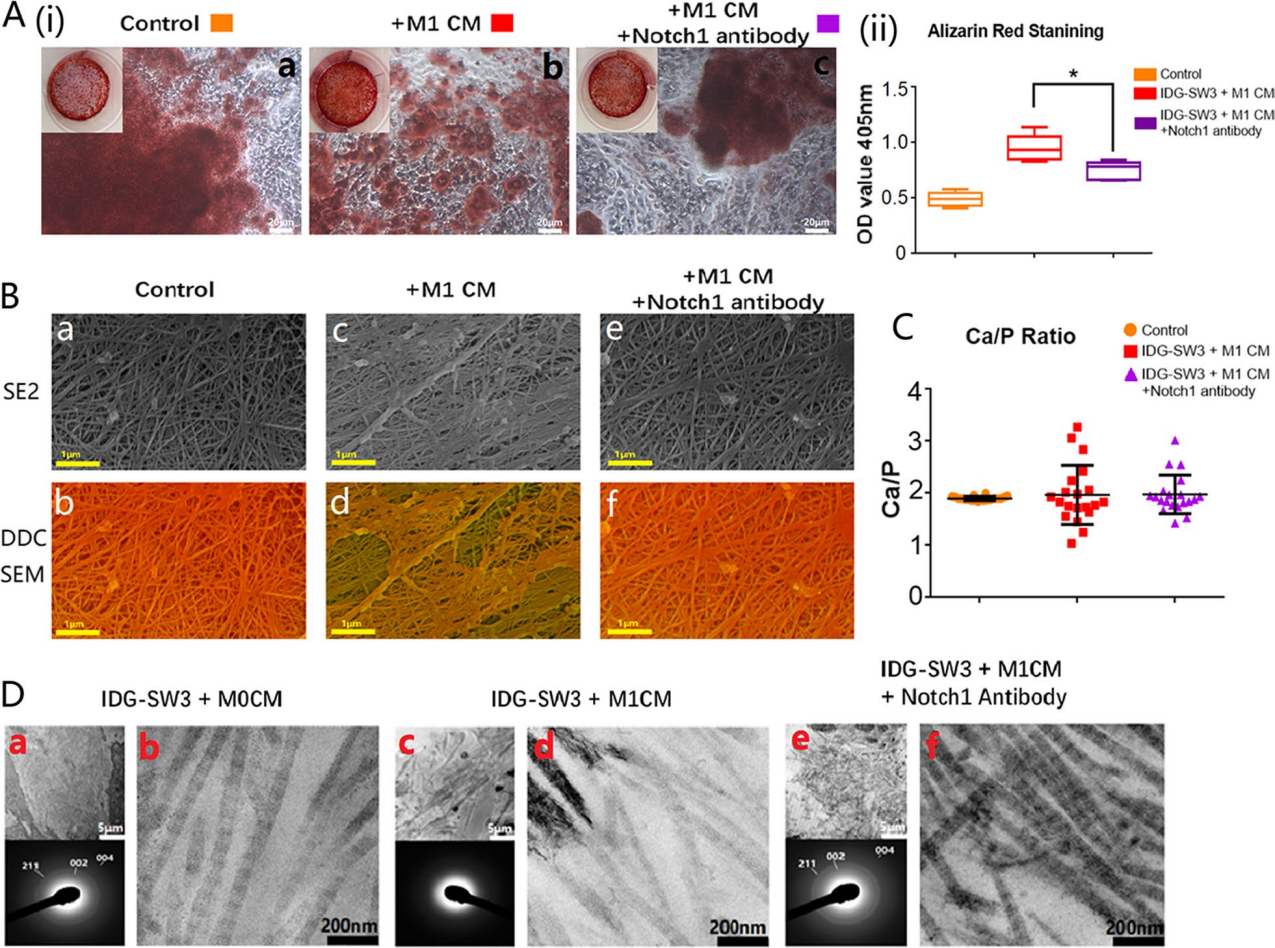
Activation of Notch signaling reversed the negative effects of M1 macrophages on osteocyte mineralization. **A**(i) Alizarin Red S staining images of M1 macrophage-stimulated osteocytes with or without Notch activation; **A**(ii) the bar graph showed the average OD value of Alizarin Red S staining; OD values were shown as mean ± SD (*p < 0.05, one-way ANOVA); **B** SEM images of mineralized collagen fibers synthesized from M1 macrophage-stimulated osteocytes with or without activation of Notch signaling pathway (Top: images captured by SE2 mode; Bottom: DDC-SEM images; scale bars represented 1 μm); **C** Ca/P ratio of mineralized collagen from the above samples detected with EDS; **D** TEM images and X-ray diffraction patterns demonstrated the ultrastructure of mineralized collagen synthesized by macrophage-stimulated osteocytes with or without Notch signaling pathway activation

The M1 macrophage-stimulated osteocytes with or without Notch signaling activation were stained with Alizarin Red S on day 14 to visualize calcium compound formation. The representative images identified that osteocytes cultured with Notch activation (Fig. 7A(i)—c) showed a decreased amount of mineralized nodule formation after culture with M1 macrophage-derived CM compared with the M1 macrophage-treated samples without Notch activation (Fig. 7A(i)—b) which was further proven by presenting decreased intensity compared with M1 macrophage-treated samples detected with the microplate absorbance spectrophotometer. However, high magnification showed the morphology of mineralized nodules formed from M1-CM+ anti-Notch 1 antibody treated osteocytes were of a larger size and distributed more evenly, similar to the control group (Fig. 7A(i)—a), compared to the M1-CM treated group.

### Activation of Notch signaling reversed the negative effects of M1 macrophages on collagen fiber mineralization

The DDC-SEM results identified the density distribution of mineralized collagen from M1-CM stimulated osteocytes (Fig. 7B(c, d)) to be lower and uneven compared to the control group (Fig. 7B(a, b)). However, the distribution of mineral deposits was more even in the Notch activated group and comparable to the control group (Fig. 7B(e, f)). Twenty randomly selected points on mineralized collagen fibers from these samples, were detected individually with EDS. The average Ca/P ratio was around 1.90 in the M1-CM-treated, and untreated samples. The Ca/P ratio mean value of both M1-CM-treated samples with or without Notch activation fluctuated, yet the mean values of M1-CM-treated samples with Notch activation were more consistent than those without Notch activation (Fig. 7C).

### Activation of Notch signaling reversed the negative effects of M1 macrophages on the ultrastructure of osteocyte mineralization

M1 macrophages could negatively affect the integration of minerals into collagen, showing less distinctive black-white bands that could be observed on mineralized collagen fibers in the TEM images (Fig. 7D(d)). Additionally, spontaneous mineral aggregations were also found in M1 macrophage-stimulated osteocytes (Fig. 7D(c)). After Notch activation, the black-white bands were clearly visible via TEM images (Fig. 7D(f)), revealing that minerals were able to be transported into the collagen gap zones. Distinctive rings of normal mineralization located at (211), (004), and (002) were present through X-ray diffraction (Fig. 7D(e)), indicating that the negative effects of M1 macrophages on the mineralized collagen ultrastructure were reversed by Notch activation.

## Discussion

The interplay between macrophages and osteocytes is critical for the maintenance of bone health yet few research has investigated the interrelation and relevant mechanisms especially in the context of inflammatory bone remodeling. Our study is the first to demonstrate inflammatory M1 macrophages prevent the maturation of osteocytes. The immaturity of osteocyte could be observed from its abnormal morphology, which appeared as a round shape. Mature osteocytes have a spindle-like cell body with a cytoplasmic dendritic structure (Bonewald 2011), whereas osteocytes at an immature stage, called pre-osteocyte or osteoid osteocyte, normally present as a round shape (Franz-Odendaal et al. 2006). Hence, the morphological change suggested that osteocyte maturation was affected in inflammatory conditions, which was accompanied with the high level of E11 and low level of DMP1. E11 is associated with osteocyte morphological construction and produced in the immature stage (Zhang et al. 2006; Moharrer and Boerckel 2021). DMP1 is a mature osteocyte marker, which transports calcium salts during the mineralization process, without which minerals cannot infiltrate the gap zone of collagen fibers to form a crystal structure (Feng et al. 2006; Qin et al. 2007). This results in the function difference between mature and immature osteocytes (Bonewald 2011). Hence, our finding indicates that M1 macrophages hinder osteocyte maturation, which might also affect mineralization.

Further investigations were performed to detect the influence of inflammation on mineralization. Bone mineralization is a highly controlled process that combines inorganic calcium salts with organic collagen fibers to form a crystal structure (Qin et al. 2007). The bone collagen molecules are staggered to form a collagen fiber, which leaves isometric gap zones between each other to allow minerals to fill in, a DMP1-dependant process (Jager and Fratzl 2000). The ultrastructure of a healthy bone shows calcium phosphates infiltrated the gap zone of collagen fibers, called crystal hydroxyapatite (Jager and Fratzl 2000). These mineral-infiltrated collagen fibers presented as crossed fibers with black and white bands, with the black bands representing the mineral-bonded gap zones (Jager and Fratzl 2000). In the present study, M1-CM induced more mineralized nodules with unevenly distributed collagen fibers, substantial variability of the Ca/P values, and especially no black-white band ultrastructure, suggesting the abnormal mineralization quality, which was further demonstrated by the different diffraction patterns from normal mineralization. This suggests that M1 macrophages prevent osteocytes forming the crystallized structure of mineralized collagen, as due to the lack of DMP1, minerals cannot infiltrate well into collagen fibers under such environment.

The negative effects of M1 macrophages on osteocyte maturation/mineralization were further observed in a rat model of OA, an inflammatory disease with subchondral bone deformities. Inflammatory subchondral osteocytes displayed similar morphological changes as our in vitro finding, suggesting the immaturity of these osteocytes, as verified by the increased E11-expression and reduced DMP-1-expression compared with osteocytes from normal subchondral bone. Accordingly, mineralization quality of OA subchondral bone showed unevenly distributed density and distinctly distributed Ca/P ratio. Along with the abnormal osteocytes/bone quality was the increased infiltration of M1 macrophages in the marrow area of inflammatory OA subchondral bone, which located adjacent to the E11^+^ immature osteocytes. Based on the above in vitro and in vivo findings, it is speculated that under inflammatory conditions, the crosstalk between M1 macrophages and differentiating osteocytes results in restricted maturation of osteocytes, which eventually leads to abnormal bone mineralization.

We then sought to investigate the mechanisms underlying the disturbed osteocyte maturation. Researchers found that suppressing Notch in osteocytes prevents osteocytes reaching maturity and negatively affects the mineralization, suggesting that Notch plays an essential role in osteocyte maturation (Shao et al. 2018a). The Notch signaling pathway is highly activated in undifferentiated MSCs (Shao et al. 2018a), which is down-regulated during differentiation towards osteoblasts (Shao et al. 2018a), and then re-upregulated during the differentiation from late-stage osteoblasts to mature osteocytes (Shao et al. 2018a). However, the function of Notch signaling pathway is different in MSCs and osteocytes. Notch signaling pathway is responsible for cell proliferation in MSCs, but determines cell maturation in osteocytes (Liu et al. 2016a). Recent research points out that HES1, a Notch targeted gene, plays a vital role in osteocyte maturation (Shao et al. 2018a; Zanotti et al. 2011; Liu et al. 2016a). It was therefore speculated that Notch signaling pathway could be involved in the M1 macrophage-stimulated osteocyte immaturity. As the target gene controlled by Notch, HES1 is a recognized factor responsible for osteocyte maturation (Shao et al. 2018a; Zanotti et al. 2011; Zhang et al. 2016b; Liu et al. 2016b), which was significantly down-regulated in pre-osteocytes treated with M1-CM. Similarly, in inflammatory subchondral bone, the expression of HES1^+^ osteocytes was obviously reduced compared with the normal bone, and HES1^+^ osteocytes distributed around CD86^+^ M1-like cells. These findings suggested that M1 macrophages could inhibit osteocyte maturation and mineralization via suppressing the Notch signaling pathway in osteocytes.

To confirm this point, Notch signaling pathway was re-activated in M1 macrophage CM-treated osteocytes using Notch antibody-coated cell culture plate, which is a well-established method (Conboy et al. 2003; Shao et al. 2018b). Upon Notch activation, osteocytes transformed from the round shape back to a spindle-like shape, and showed an increased DMP1 and a reduced E11 expression, suggesting Notch activation turned these osteocytes into a more mature state. This eventually corrected the abnormal mineralization, as shown by the even density distribution of the mineralized collagen and evenly distributed Ca/P ratios, due to the recovered DMP1 expression. Furthermore, this promoted the infiltration of calcium phosphates into the collagen fiber gap zone, showing isometric black-white bands on collagen fiber bundles, with diffraction rings reappearance, indicating that M1 macrophage-stimulated abnormal mineralized collagen crystallization process was corrected by Notch activation in osteocytes. Therefore, it is concluded that under inflammatory conditions, M1 macrophages secrete factors to disturb osteocyte maturation via inhibiting the Notch signaling pathway, which eventually results in abnormal bone mineralization. Furthermore, osteocytes at such immature stage might facilitate osteoclastogenesis, as immature osteocytes expressed increased RANKL and reduced OPG (Additional file 1: Fig. S1), facilitating the differentiation of osteoclasts (Aliprantis et al. 2016; Ponzetti and Rucci 2019), thereby further contributes to the subchondral bone abnormities.

Previous studies have found that M1 inflammatory macrophages facilitate osteogenic differentiation and mineralization, showing a much stronger osteoinductivity than M0/M2 phenotypes. Interestingly, M1 to M2 transition has been confirmed in natural bone healing, especially, biomaterials facilitating this transition could improve bone regeneration (Chen et al. 2016). These paradoxical findings could be partially explained by the present study, that although M1 macrophages seem to induce mineralization, the minerals formed under such condition are unqualified compared with normal ones, which might be due to the impaired osteocyte maturation. The major cytokines contained in M1-CM or M2-CM were detected using ELISA. The most common pro-inflammatory cytokines, such as IL-1, IL-6, and TNF-α were found in M1-CM, while the anti-inflammatory cytokines were found in M2-CM (as shown in Additional file 5: Fig. S5). It is believed that cytokines secreted by macrophages play important roles in regulating osteocyte maturation. Previous studies have demonstrated that TNF-α inhibits Notch-1 in skeletal muscle cells, suggesting that M1 macrophages might directly suppress Notch signaling pathway in osteocytes via secreting cytokines within the inflammatory microenvironment (Acharyya et al. 2010). Exosomes released from M1 macrophages containing microRNAs, such as miR-34s and miR-146 might also participate in inhibiting the Notch-mediated osteocyte maturation (Wei et al. 2019; Bae et al. 2012; Huang et al. 2016). Furthermore, M1 macrophage-stimulated Notch inhibition might in turn facilitate the activation of Wnt signaling pathway (Shao et al. 2018b), a key signaling pathway in early differentiation of osteoblasts. Additionally, inflammatory cytokines (as shown in Additional file 5: Fig. S5), such as IL-1 and TNF-α have been reported to induce Wnt signaling pathway (Briolay et al. 2013; Yoshida et al. 2018), while growth differentiation factor 15 (GDF-15) has been reported to induce both MAPK and mTOR signaling pathway (Griner et al. 2013). Activating Wnt, MAPK, or mTOR signaling pathway can result in Notch deactivation (Shao et al. 2018b; Zeng et al. 2005; Sun et al. 2012), indicating that inflammatory macrophages can also indirectly suppress Notch signaling pathway in osteocytes. Accordingly, we found that M1-CM resulted in decreased expression of SOST (as shown in Additional file 1: Fig. S1), a mature osteocyte-derived factor which prevents the differentiation of osteoblasts. Since SOST expression is inactivated by the Wnt signaling pathway (Tu et al. 2012; Zhou et al. 2019), the decreased SOST expression could be attributed to M1 macrophage-stimulated Notch inhibition. It is therefore speculated that inflammatory microenvironment, which inhibits Notch while induces Wnt, is beneficial for early-stage osteogenic differentiation towards osteoblasts. However, at the late stage of osteogenesis, the inflammatory microenvironment is not preferred, as the continuous inhibition on Notch signaling pathway can hinder the maturation of osteocytes and thereby result in abnormal mineralization. M2 macrophage-derived conditioned medium did not show any significant effects on osteocyte maturation and mineralization in our study. However, three major M2 macrophage-derived cytokines were detected from the conditioned medium including platelet-derived growth factor BB (PDGF-BB), interleukin 33 (IL-33), and growth arrest specific 6 (Gas6) (Additional file 5: Fig. S5Aii), which can regulate bone remodeling. PDGF-BB acts as an enhancer on early MSC differentiation towards osteoblasts via increasing cellular ALP level (Zhang et al. 2021). IL-33 prevents osteoclast differentiation via inhibiting TNF-α expression (Ohori et al. 2020). The M2-macrophage-derived cytokines may therefore affect osteocyte activities and is worthy of further exploration.

Research into osteocytes requires culturing large numbers of osteocytes, but osteocytes are a kind of terminally differentiated cells that do not proliferate during culture (Shah et al. 2016). Using primary osteocytes means a large number of animals will be sacrificed to obtain sufficient osteocytes. IDG-SW3, a novel murine immortal osteocyte cell line, is the most suitable cell for in vitro cell model building, as it can represent the maturation process of osteocytes and mineralization (Woo et al. 2011). Comparing to other osteocyte cell lines (HOB-01-C1, MLO-A5, and MLO-Y4) representing early osteoblast differentiation process (Kato et al. 1997; Kato et al. 2001; Bodine et al. 1996), IDG-SW3 demonstrated to represent the terminally differentiated osteocytes, and the typical osteocyte markers such as E11, DMP1, and SOST are detectable in IDG-SW3 cells (Woo et al. 2011). This cell line has a code of green fluorescence protein (GFP) gene attached at the end of the DMP1 gene, whose green fluorescence is detectable once DMP1 expressed (Woo et al. 2011). Additionally, IDG-SW3 is originally from the same specie as the monocyte/macrophage-like cell line used in this study.

## Conclusions

**Fig. 8:**
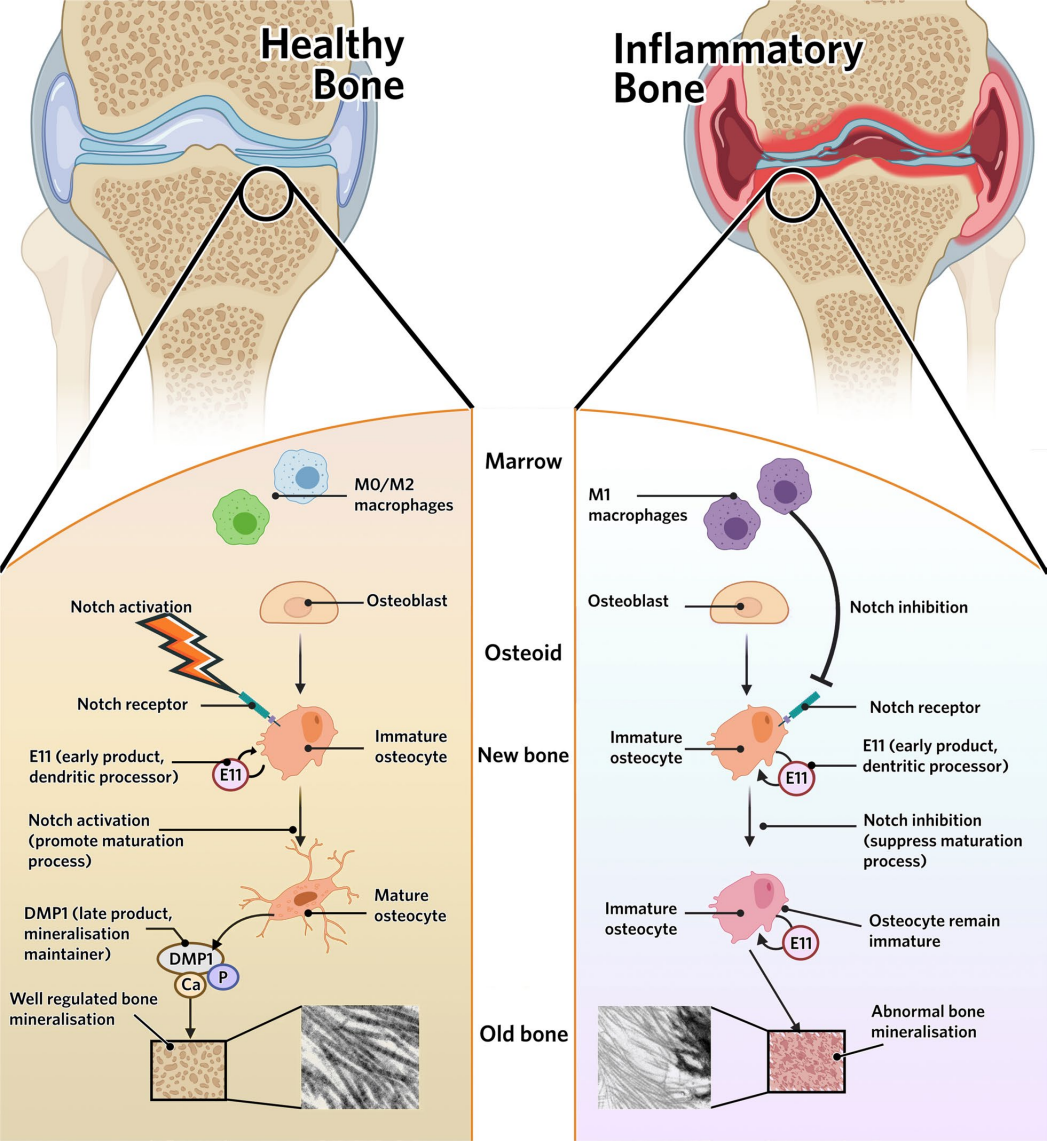
Inflammatory M1 macrophage negatively affects osteocyte maturation and mineralization via inhibiting Notch signaling pathway. Osteocyte maturation requires activation of the Notch signaling pathway. Only mature osteocytes produce DMP1 which is critical in bone mineralization. DMP1 is the transporter of calcium phosphate, only with which minerals and collagen fibers combine regularly to form well crystallized bone structure. Inflammatory M1 macrophages inhibit Notch signaling pathway in osteocytes, preventing osteocytes reaching maturity. Immature osteocytes cannot produce DMP1. Lack of DMP1 calcium phosphate cannot be transported into collagen gap zones, therefore minerals cannot integrate into collagen fibers in an organized manner and calcium deposition is formed. Disorganized bone mineralization finally forms abnormal bone structure (Schematic figure created with BioRender.com)

Taken together, this study demonstrated the role of macrophage-osteocyte crosstalk in bone mineralization (Fig. 8). Especially in inflammatory conditions, immune cells such as M1 macrophages impede the maturation of osteocytes, which results in abnormal bone mineralization. This study also reveals that Notch could be a potential therapeutic target for correcting osteocyte immuration in inflammatory conditions, thereby avoiding bone mineralization deformities.

## Supplementary Information

**Additional file 1: Figure S1.** Osteocytes secreted regulatory factors after macrophage simulation. The RANKL expression level in M1 macrophage-stimulated osteocytes increased while OPG decreased. Mature osteocyte-specific product SOST was down regulated by M1 macrophages. The protein levels of E11 and DMP1 expressed by M0, M1, or M2 macrophage-stimulated osteocytes were detected by western blot, α-Tubulin was used as an internal control. Data from three independent experiments were shown as mean ± SD (*p < 0.05, one-way ANOVA).

**Additional file 2: Figure S2.** Isotype control of IHC staining of normal and inflammatory bone. Normal and inflammatory bone sections stained with H&E (a and e: low magnification, the scale bars represented 100 μm; b and f: high magnification, the scale bars represented 20 μm); Normal and inflammatory bone sections stained with mouse IgG as isotype control (c and g: low magnification, the scale bars represented 40 μm; d and h: high magnification, the scale bars represented 20 μm).

**Additional file 3: Figure S3.** Double staining of normal and inflammatory bone remodeling areas confirmed the phenotype of activated macrophages. Immunofluorescence double staining of macrophage markers on (a and c) normal and (b and d) OA bone sections (CD86 and iNOS: M1 macrophage markers; CD206 and CD163: M2 macrophage markers; the scale bars represented 20 μm).

**Additional file 4: Figure S4.** Notch signaling pathway was successfully induced in M1 macrophage-stimulated osteocytes. A: Western blot results of HES1 protein expression level from osteocytes stimulated with macrophage-derived conditioned medium and Notch signaling pathway activation; B: Gene expression of HES1 from osteocytes stimulated with macrophage-derived conditioned medium and Notch signaling pathway activation; Data from three independent experiments were shown as mean ± SD (*p < 0.05, one-way ANOVA).

**Additional file 5: Figure S5.** Various cytokines secreted from M1 and M2 macrophages. M1 macrophages released relatively more proinflammatory cytokines into the conditioned medium than M2 macrophages. IL-1, IL-6, and TNF-α were the major proinflammatory cytokines contained in the M1 macrophage-derived conditioned medium. Data from three independent experiments were shown as mean ± SD (*p < 0.05, one-way ANOVA).

## Data Availability

Not applicable.

## Abbreviation

OA: Osteoarthritis
MSCs: Mesenchymal stem cells
SOST: Sclerostin
RANKL: Receptor activator of nuclear factor-κ B Ligand
OPG: Osteoprotegerin
E11: Podoplanin
Dmp1: Dentin matrix protein 1
HES1: Hairy and enhancer of split 1
CM: Conditioned medium
SEM: Scanning electron microscope
DDC-SEM: Density-dependent color scanning electron micrographs
EDS: Energy dispersive spectroscopy
BSEM: Backscattered SEM
TEM: Transmission electron microscope
DMEM: Dulbecco’s Modified Eagle Medium
P/S: Penicillin and streptomycin
FBS: Fetal bovine serum
LPS: Lipopolysaccharide
IFN-γ: Interferon gamma
IL-4: Interleukin 4
PBS: Phosphate buffered saline
GFP: Green fluorescence protein
AU: Arbitrary unit
qRT-PCR: quantitative reverse transcription polymerase chain reaction
H&E: Hematoxylin and eosin
TRAP: Tartrate-resistant acid phosphatase
FOV: Field of view
IHC: Immunohistochemistry
BSA: Bovine serum albumin
IgG: Immunoglobulin G
DAB: Diaminobenzidine
IF: Immunofluorescence
DAPI: 4′,6-diamidino-2-phenylindole
SD: Standard deviation
PDGF-BB: Platelet-derived growth factor BB
IL-33: Interleukin 33
Gas6: Growth arrest specific 6
IL-1: Interleukin 1
TNF-α: Tumor necrosis factor alpha
ANOVA: Analysis of variance
GDF-15: Growth differentiation factor-15
