# Supplementary figures and images for: Inflammatory macrophages interrupt osteocyte maturation and mineralization via regulating the Notch signaling pathway
Source: Mol Med. 2022 Sep 4;28:102. doi: 10.1186/s10020-022-00530-4 (PMC9441044; doi:10.1186/s10020-022-00530-4)

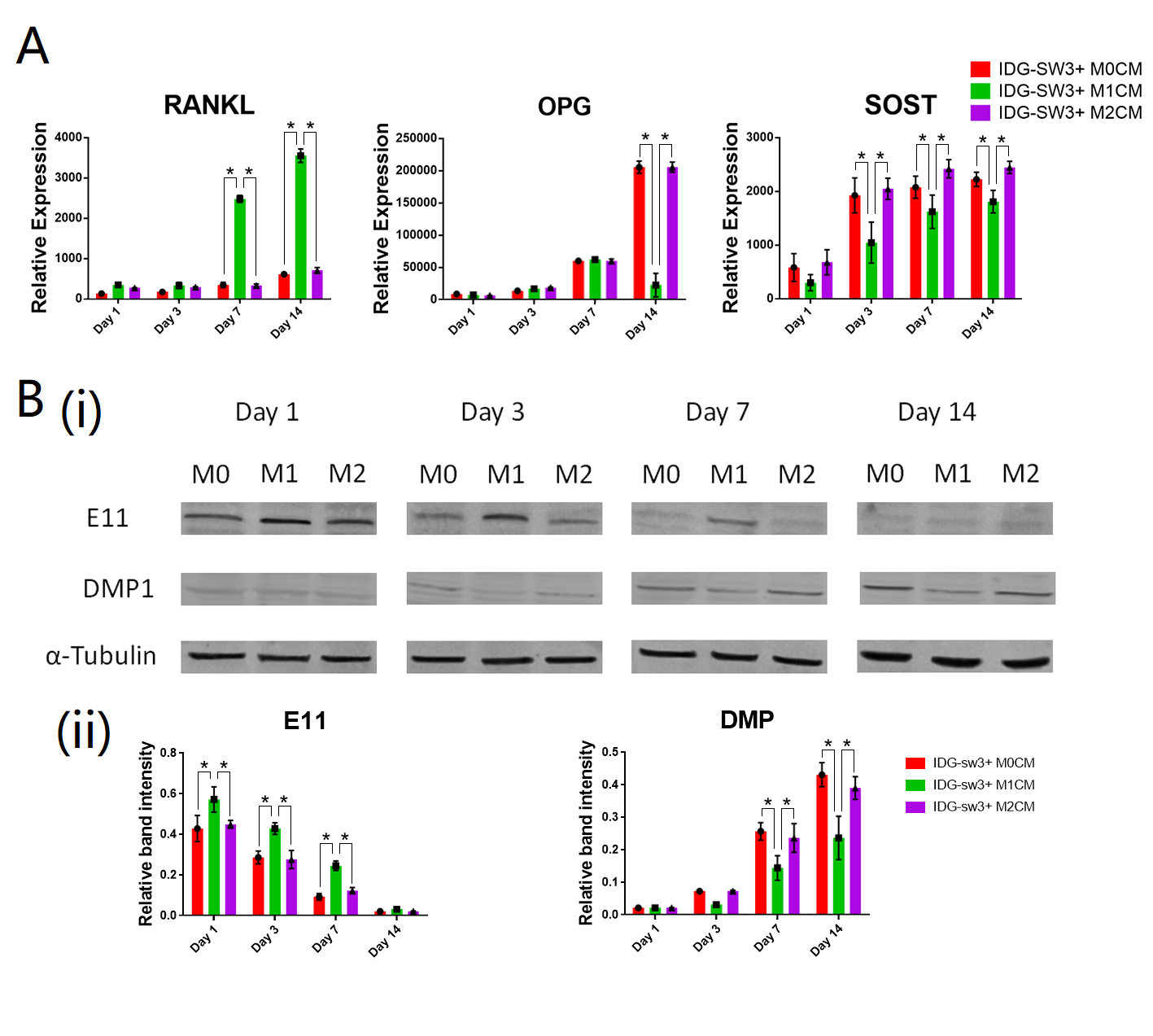

Supplement: Supplementary file 1 — Additional file 1: Figure S1. Osteocytes secreted regulatory factors after macrophage simulation. The RANKL expression level in M1 macrophage-stimulated osteocytes increased while OPG decreased. Mature osteocyte-specific product SOST was down regulated by M1 macrophages. The protein levels of E11 and DMP1 expressed by M0, M1, or M2 macrophage-stimulated osteocytes were detected by western blot, α-Tubulin was used as an internal control. Data from three independent experiments were shown as mean ± SD (*p < 0.05, one-way ANOVA). [file 10020_2022_530_MOESM1_ESM.tif]

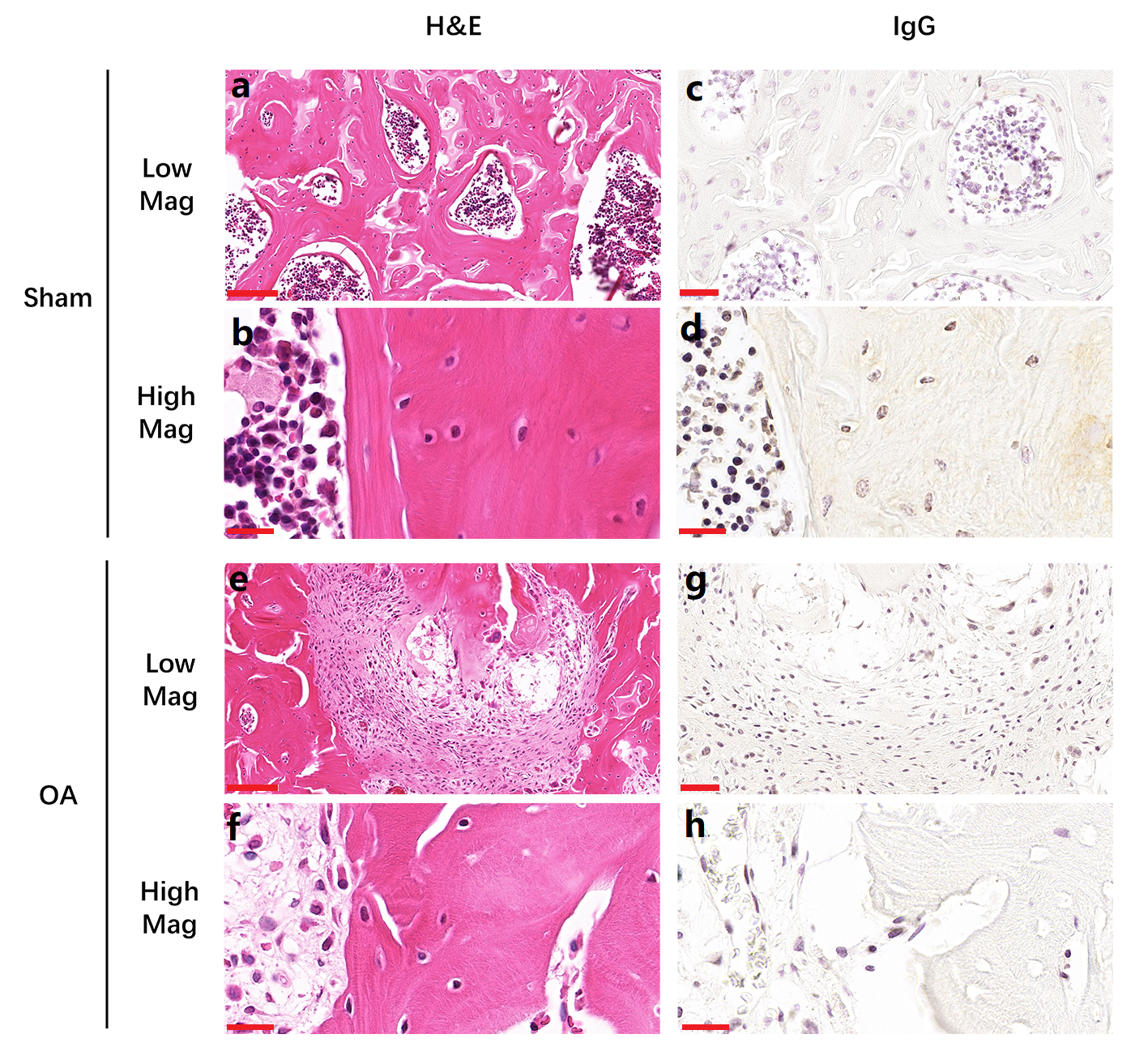

Supplement: Supplementary file 2 — Additional file 2: Figure S2. Isotype control of IHC staining of normal and inflammatory bone. Normal and inflammatory bone sections stained with H&E (a and e: low magnification, the scale bars represented 100 μm; b and f: high magnification, the scale bars represented 20 μm); Normal and inflammatory bone sections stained with mouse IgG as isotype control (c and g: low magnification, the scale bars represented 40 μm; d and h: high magnification, the scale bars represented 20 μm). [file 10020_2022_530_MOESM2_ESM.tif]

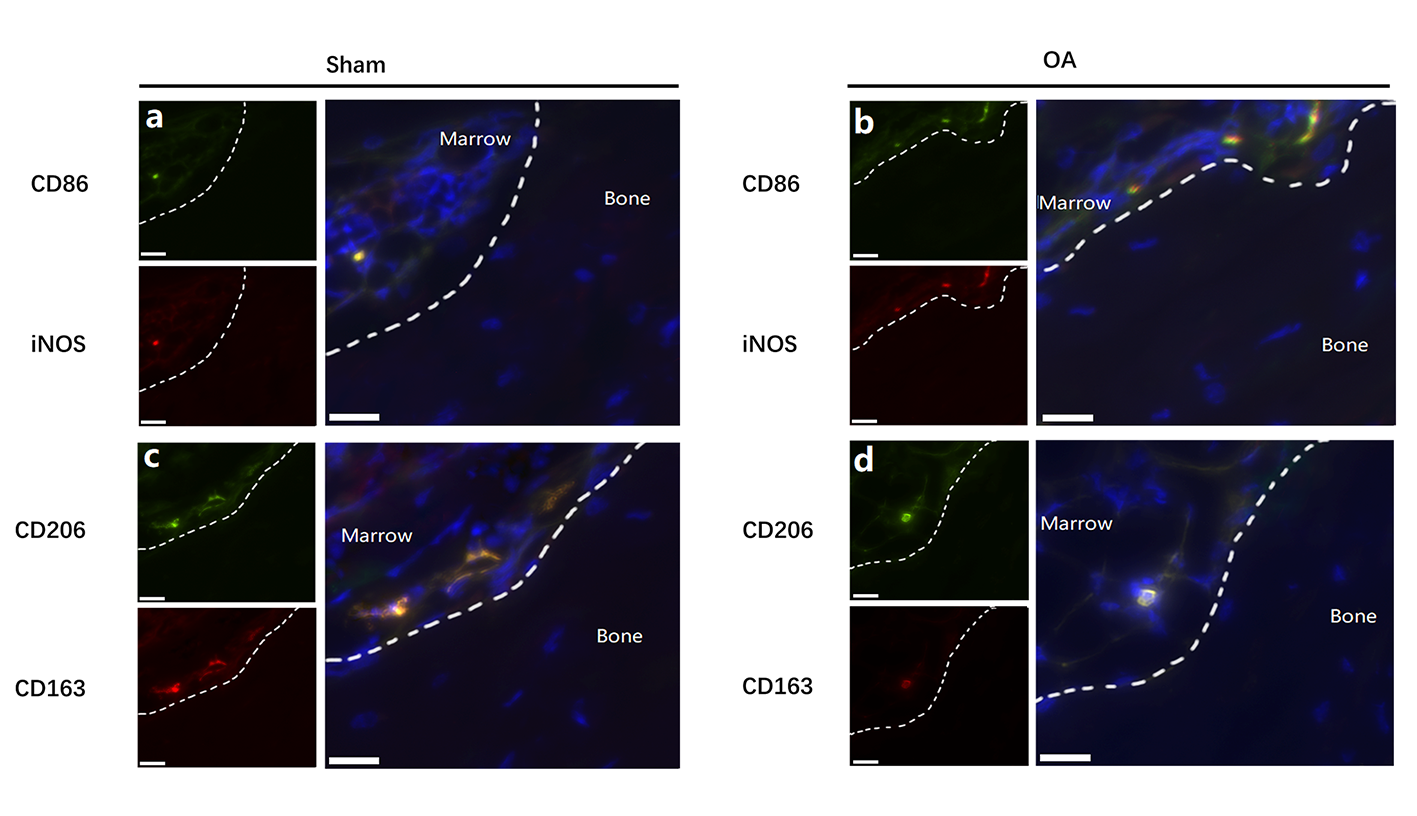

Supplement: Supplementary file 3 — Additional file 3: Figure S3. Double staining of normal and inflammatory bone remodeling areas confirmed the phenotype of activated macrophages. Immunofluorescence double staining of macrophage markers on (a and c) normal and (b and d) OA bone sections (CD86 and iNOS: M1 macrophage markers; CD206 and CD163: M2 macrophage markers; the scale bars represented 20 μm). [file 10020_2022_530_MOESM3_ESM.tif]

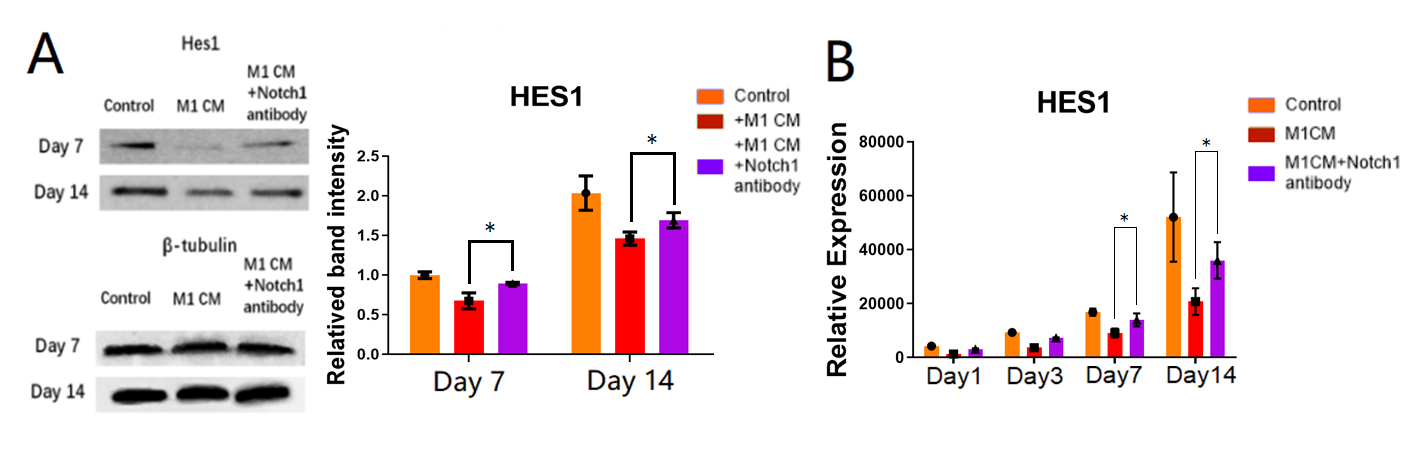

Supplement: Supplementary file 4 — Additional file 4: Figure S4. Notch signaling pathway was successfully induced in M1 macrophage-stimulated osteocytes. A: Western blot results of HES1 protein expression level from osteocytes stimulated with macrophage-derived conditioned medium and Notch signaling pathway activation; B: Gene expression of HES1 from osteocytes stimulated with macrophage-derived conditioned medium and Notch signaling pathway activation; Data from three independent experiments were shown as mean ± SD (*p < 0.05, one-way ANOVA). [file 10020_2022_530_MOESM4_ESM.tif]

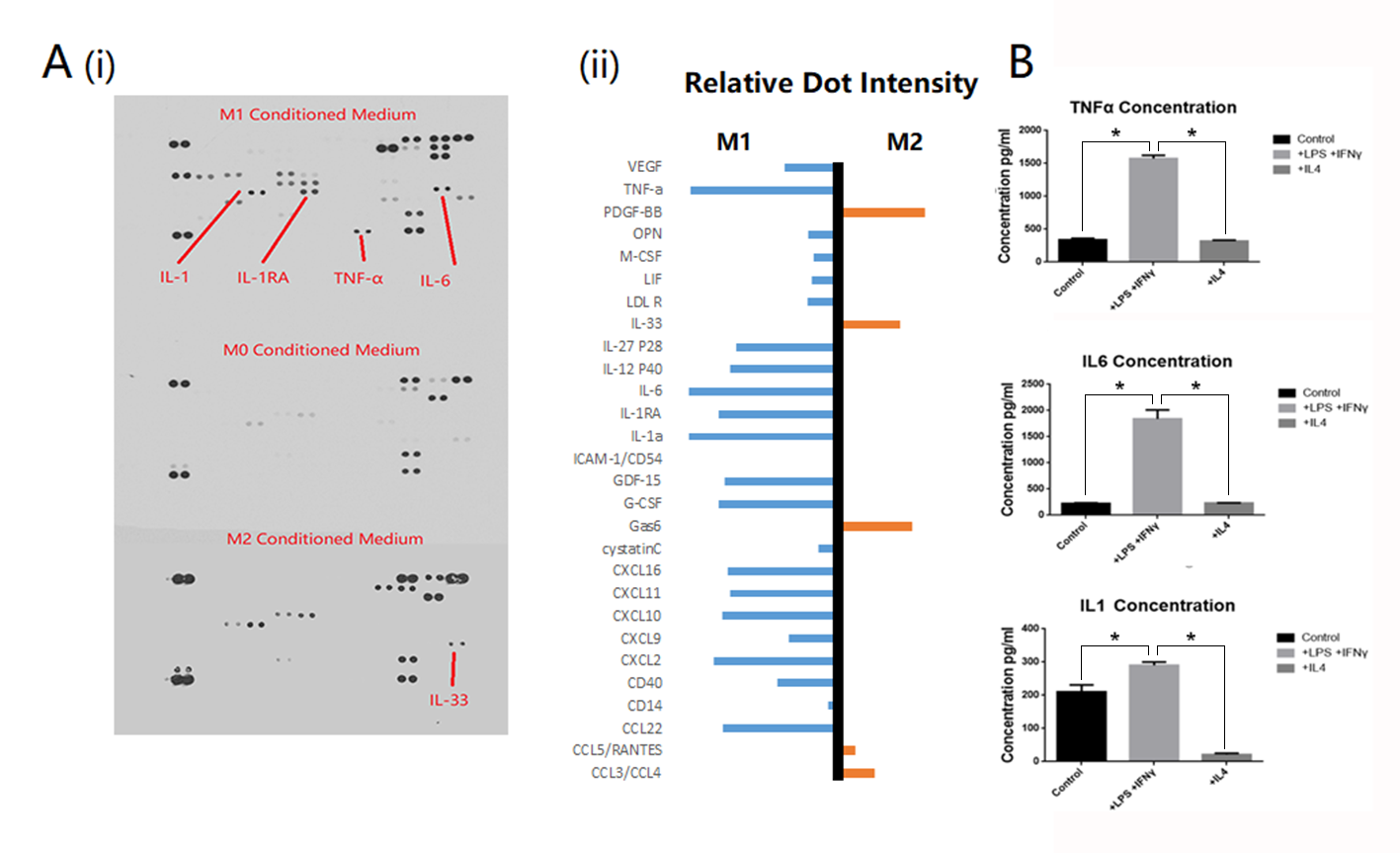

Supplement: Supplementary file 5 — Additional file 5: Figure S5. Various cytokines secreted from M1 and M2 macrophages. M1 macrophages released relatively more proinflammatory cytokines into the conditioned medium than M2 macrophages. IL-1, IL-6, and TNF-α were the major proinflammatory cytokines contained in the M1 macrophage-derived conditioned medium. Data from three independent experiments were shown as mean ± SD (*p < 0.05, one-way ANOVA). [file 10020_2022_530_MOESM5_ESM.tif]
